# Robust Atomistic Modeling of Materials, Organometallic, and Biochemical Systems

**DOI:** 10.1002/anie.202004239

**Published:** 2020-05-18

**Authors:** Sebastian Spicher, Stefan Grimme

**Affiliations:** ^1^ Mulliken Center for Theoretical Chemistry University of Bonn Beringstr. 4 53115 Bonn Germany

**Keywords:** computational chemistry, dynamics, force-field, molecular modeling, molecular structures

## Abstract

Modern chemistry seems to be unlimited in molecular size and elemental composition. Metal‐organic frameworks or biological macromolecules involve complex architectures and a large variety of elements. Yet, a general and broadly applicable theoretical method to describe the structures and interactions of molecules beyond the 1000‐atom size regime semi‐quantitatively is not self‐evident. For this purpose, a generic force field named GFN‐FF is presented, which is completely newly developed to enable fast structure optimizations and molecular‐dynamics simulations for basically any chemical structure consisting of elements up to radon. The freely available computer program requires only starting coordinates and elemental composition as input from which, fully automatically, all potential‐energy terms are constructed. GFN‐FF outperforms other force fields in terms of generality and accuracy, approaching the performance of much more elaborate quantum‐mechanical methods in many cases.

## Graphical abstract

**Gotta catch ′em all**: A completely automated, partially polarizable, generic force field for the accurate description of structures and dynamics of large molecules across the periodic table is presented. This method, termed GFN‐FF, combines force‐field speed with almost quantum‐mechanical accuracy.

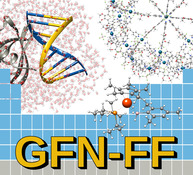

## Introduction

Concepts for designing molecules with desired (bio)chemical activities or physical properties have become state‐of‐the‐art in experimental chemistry.^1^, ^2^ Molecular size and complexity has no boundaries and the elemental composition is versatile.^3^ Within the last decades, the field of theoretical chemistry has evolved into an indispensable part of chemistry and has proven to be an important companion of the experiment.^4^ Computational chemistry is able to explore the chemical space and provide experimentalists with useful information in order to circumvent resource‐demanding trial‐and‐error procedures.^5^, ^6^ In a cleaner and greener future for chemistry, theory is an essential tool supporting the experiment and increasing economic and environmental sustainability.^7^ The constantly growing diversity of chemical‐compound space requires the development of new methods that can be applied in the analysis and prediction of complex molecular systems. Yet, a universal, fast, and easy‐to‐use method that is capable of providing qualitatively correct molecular models beyond the size of a thousand atoms with arbitrary elemental composition is missing.^8^

Even though today's ensemble of theoretical methods is quite versatile, it is limited in application. On the basis of wave‐function theory (WFT), methods have been developed that can provide highly accurate total energies and potential‐energy surfaces (PES) for small to medium‐sized molecules in the gas phase.^9^ Kohn–Sham density functional theory (KS‐DFT or simply DFT) draws the connection between the energy of a system and its electron density. With the introduction of reasonable approximations, DFT methods can routinely provide accurate PES for systems with up to a few hundred atoms.^10^ Latest developments in the field of semiempirical quantum‐mechanical (SQM)^11^, ^12^ methods have further extended the treatable molecular size with special attention regarding the computation of geometries, frequencies, and non‐covalent interactions (GFN).^13^, ^14^ Within the extended tight‐binding (xTB) theoretical framework, equilibrium‐structure optimizations and molecular‐dynamics (MD) simulations are feasible for large molecular systems, aiming at a comparable accuracy as DFT.^15^ Still, the routine handling of several thousands of atoms is beyond the scope for the aforementioned methods and it is therefore necessary to apply more drastic but still physically reasonable approximations to reduce computational demands.^16^

**Figure 1:**
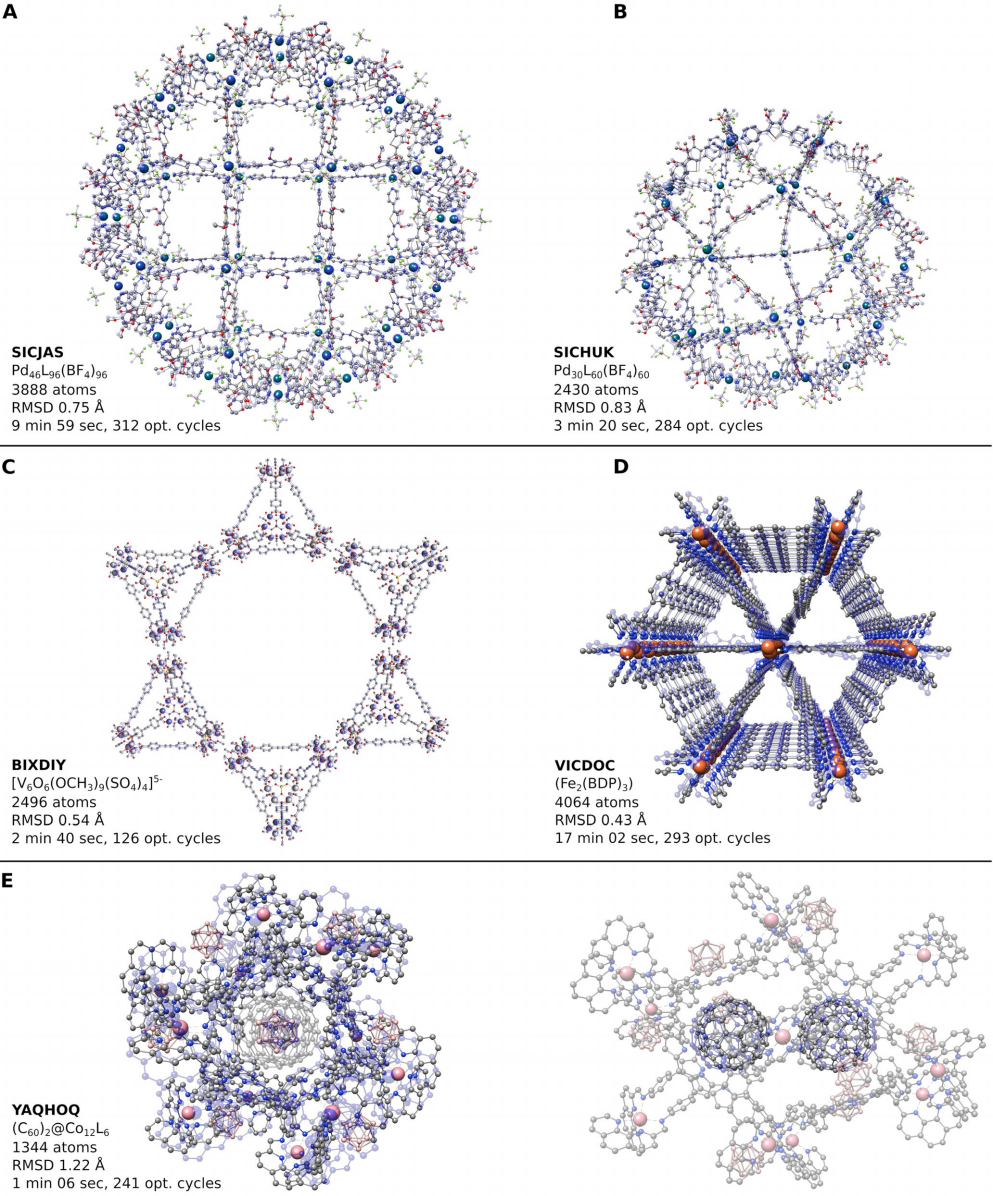
Modeling of metal‐organic porous materials with GFN‐FF. A)–E) RMSD‐minimized structure overlay between the optimized GFN‐FF geometries (transparent blue) and crystal structures of the five systems. The CSD identifiers are given as well as heavy‐atom RMSD values, total computation wall‐times, and the required number of geometry‐optimization cycles.

Neglecting the electronic structure of a molecule and replacing it by classical interatomic interaction potentials is the main approximation in classical, atomistic force fields (FFs). Their great benefit is to leave out the costly and difficult description of the electronic structure and substituting it by chemical‐knowledge‐motivated classical energy expressions. FFs specialized for the accurate description of a certain class of chemical systems exist for various fields of application. Organics are well described by GAFF and MM3,^17^ while CHARMM,^18^, ^19^ Amber,^20^ and OPLS^21^ focus on the description of proteins. In materials science, DREIDING^22^ and MOF‐FF^23^ are widely used. Limitations of those special‐purpose FFs are manifold, as they are not suited for interdisciplinary use, given the fact that parameters only exist for a limited amount of elements and structural motifs. Until now, only a single general applicable FF covering a full periodic‐table parameterization exists. This universal force field (UFF)^24^ was first introduced in 1992 and ever since, advancements on this subject could not prevail, requiring, most of the time, individual laborious parameterizations. Within this work, the idea of a general, easy‐to‐use force field is revived within the GFN framework. The presented method named GFN‐FF represents a generic, fully automated potential for the accurate description of an unlimited variety of molecular systems. GFN‐FF is designed to combine high force‐field speed with the accuracy of QM methods at unsurpassed robustness. For a manifold of systems, GFN‐FF is currently the only applicable atomistic method to provide reasonable theoretical molecular structures. As examples for the diverse possible applications, a selected set of five porous metal‐organic materials is shown in Figure 1. Their discussion will follow in the results section below.

## Method

**Figure 2:**
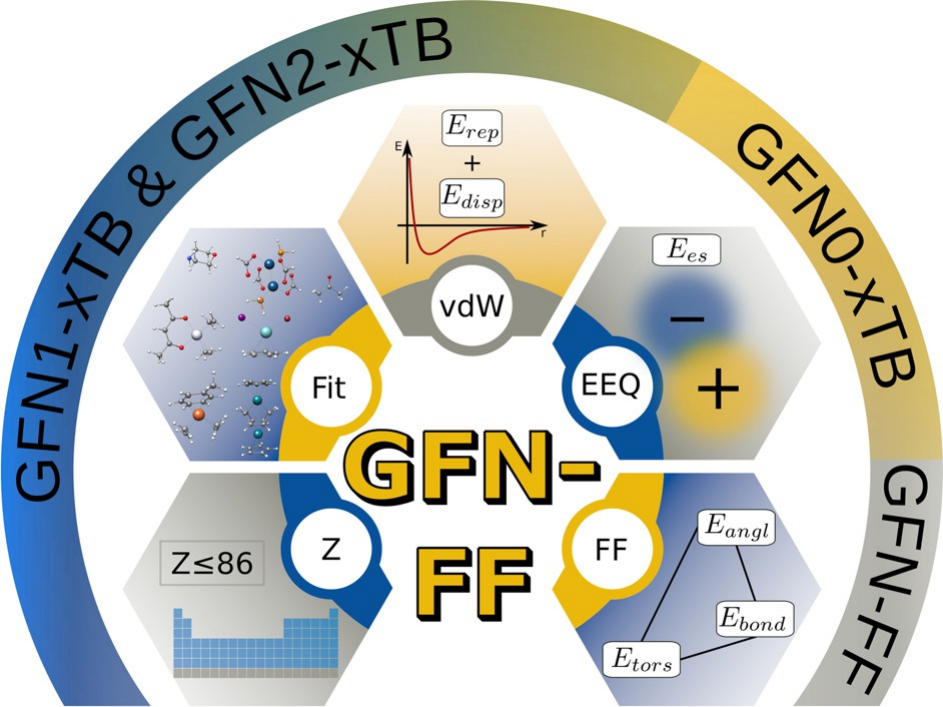
The GFN family of methods. The graphic shows the ingredients to the GFN‐FF potential energy and the connection to the family members.

The idea of a general GFN‐type FF is inspired by the latest developments in the field of SQM methods, namely the evolution of GFN1‐, GFN2, and especially GNF0‐xTB^25^ methods, where the latest key ingredient was the introduction of a classical electronegativity‐equilibrium (EEQ) atomic‐charge model^26^, ^27^ for the description of pairwise interatomic electrostatic interactions. This allowed to truncate the fundamental expansion of the DFT energy *E*[*ρ*] in terms of electron‐density fluctuations δ*ρ* after the first‐order term, leading to a non‐self‐consistent method which employs classical atomic charges. GFN‐FF introduces approximations to the remaining quantum‐mechanical terms in GFN0‐xTB by replacing most of the extended‐Hückel‐type theory (EHT) for covalent bonding by classical bond, angle, and torsion terms. To highlight the ancestry from the xTB methods, the similarities and differences between FF and QM methods are illustrated in Figure 2.

**Figure 3:**
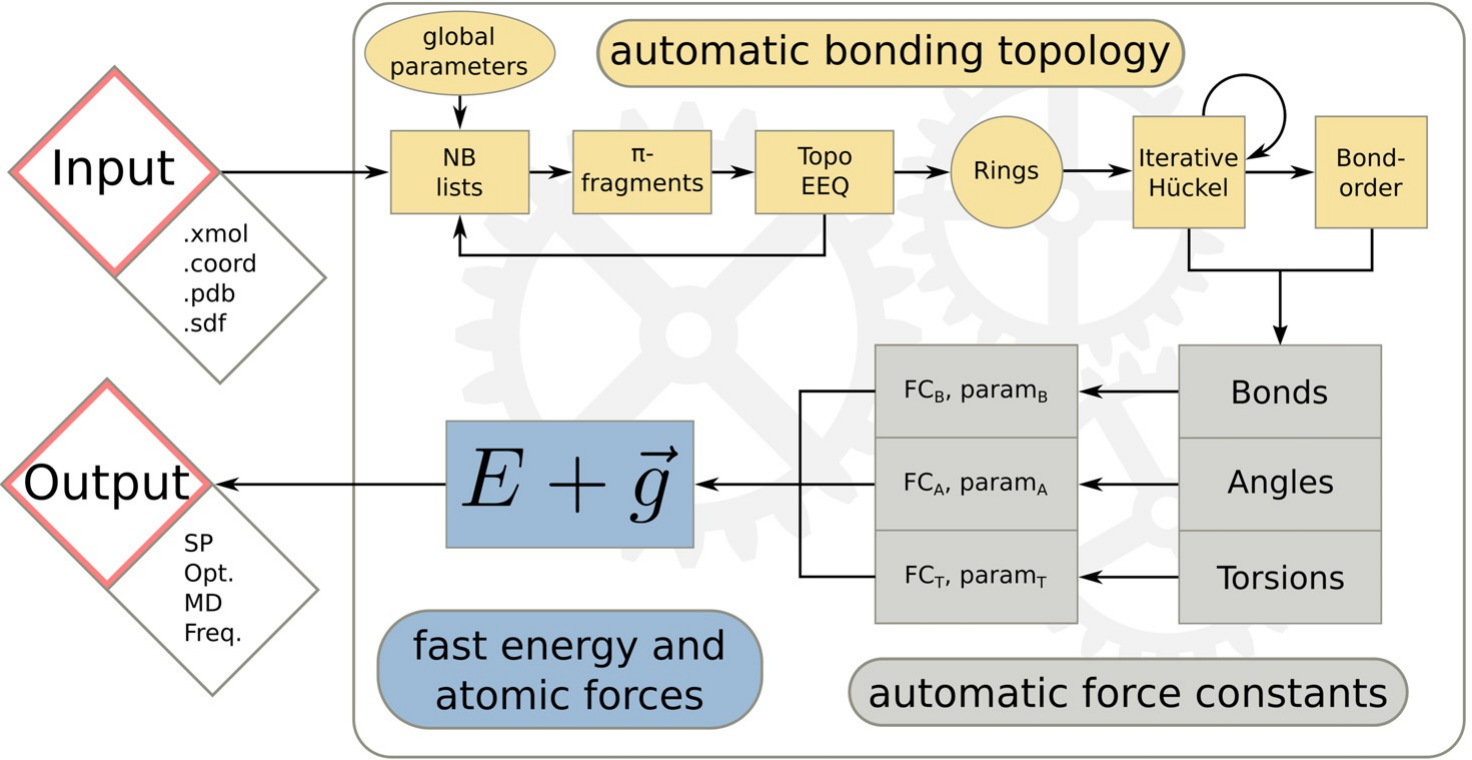
GFN‐FF internal flow chart. The implementation in the *xtb* program provides a fully automated force‐field setup, which is the generation of the topology and derivation of the force constants and other energy‐term‐related parameters.

All GFN methods cover a full‐periodic‐table parameterization for elements up to radon (*Z*≤86). This broad coverage is not self‐evident even for SQM methods. To yield accurate results, the empirical FF parameters are fitted to reproduce DFT (B97‐3c^28^) equilibrium geometries and frequencies as well as theoretical‐reference non‐covalent‐interaction energies. A mostly global and element‐specific fitting strategy is applied, thereby avoiding element‐pair‐specific parameters. The molecule training set is versatile and covers currently about 8000 structures reaching from small hydrides or oxides of the respective elements to large transition‐metal complexes. This approach is a unique feature of all GFN methods and differs strongly from the parameterization strategies of other force fields.^19^, ^29^, ^30^, ^31^ The potential‐energy terms in GFN‐FF are physically based and more sophisticated than the simple, often‐used harmonic functions. Due to this well‐defined basis, parameters arise naturally from the potential‐energy terms and their number is rather small. With only 18 specific parameters per element, GFN‐FF is constructed upon a framework flexible enough to describe a vast majority of chemical systems. The quality and complexity of the potential functions determine the accuracy of GFN‐FF rather than the sheer amount of parameters. This contradicts a current trend in theoretical chemistry to solve complicated many‐body problems with a huge number of parameters, as it is done in, for example, machine learning and neural networks.^32^, ^33^ The description of van‐der‐Waals interactions represents another parallel between the GFN methods, treating London dispersion and Pauli‐exchange repulsion almost identically. To accurately treat the important π‐conjugated systems such as aromatic hydrocarbons or graphenic materials, GFN‐FF retains an iterative Hückel QM scheme for a selected set of atoms. From the resulting bond orders, the force constants and other energy‐relevant terms are derived, leading to a high accuracy for π‐conjugated molecules. The assignment of parameters and setup of force constants is key to the performance of any FF. A simplified flow chart of the automatic setup is given in Figure 3.

This automation is an unique feature of GFN‐FF. As input, only Cartesian coordinates and the elemental composition are required, from which the topological covalent‐bonding information as well as atomic charges and bond orders are generated fully automatically. With this information at hand, all potential‐energy terms are constructed. The total GFN‐FF energy expression is given by Eq. (1)

(1) $$E_{\mathrm{GFN}-\mathrm{FF}}=E_{\mathrm{cov}}+E_{\mathrm{NCI}},$$

where *E*_cov_ refers to the bonded FF energy and *E*_NCI_ describes the intra‐ and intermolecular non‐covalent interactions (NCI). A more detailed description is given in the Supporting Information. In the covalent part, interactions are described by asymptotically correct bond stretch, bond angle, and torsional terms. For the stretch term, a new Gaussian‐type potential is proposed that allows bond cleavage, thus turning GFN‐FF into a dissociative force field. Repulsive terms are added for bonded and non‐bonded interactions separately. Additionally, a new three‐body bonding correction that extends beyond the sum of pairwise interactions is included, yielding Eq. (2):

(2) $$E_{\mathrm{cov}}=E_{\mathrm{bond}}+E_{\mathrm{bend}}+E_{\mathrm{tors}}+E_{\mathrm{rep}}^{\mathrm{bond}}+E_{\mathrm{abc}}^{\mathrm{bond}}$$

In the non‐covalent part, electrostatic interactions are described by the EEQ model. It is employed to calculate the entire electrostatic energy and isotropic atomic partial charges, which goes beyond the fixed‐charge model used in many other FFs. Overall, GFN‐FF uses two sets of EEQ charges. One set depends on the actual molecular geometry, whereas another set of charges is exclusively bond‐topology based, introducing further polarizability to the FF and leading to large simplifications for the gradient computations. Dispersion interactions are taken into account by a simplified version of the established D4 scheme,^34^ which is the most accurate dispersion correction available and superior to the corresponding description in standard FFs. Without detailed QM information, the accurate description of important non‐covalent hydrogen (and halogen) bonds (HB/XB) is challenging. Therefore, newly developed charge‐dependent HB/XB corrections are applied. These unique potentials include information about the location of electron lone‐pairs via an exclusion principle over neighboring atoms. The non‐covalent energy expression is given by Eq. (3):

(3) $$E_{\mathrm{NCI}}=E_{\mathrm{IES}}+E_{\mathrm{disp}}+E_{\mathrm{HB}}+E_{\mathrm{XB}}+E_{\mathrm{rep}}^{\mathrm{NCI}}$$

Despite its complexity, GFN‐FF reaches quadratic scaling in terms of energy‐ and gradient‐calculation time with respect to system size at a moderate prefactor and is thus not much slower than established force fields in terms of computational speed.

## Results and Discussion

To conduct chemically sensible atomistic modeling, knowledge about molecular structure, binding motifs, and structural dynamics is essential. Accurate molecular geometries give insights into the composition and functionality of the investigated system. GFN‐FF as implemented in the free *xtb* program is equipped with a highly sophisticated quasi‐Newton geometry‐optimization engine, which is also used by our QM methods. Implementation of a fragmented‐Hessian scheme provides the necessary speed‐up to be practical also at the FF level. All results discussed in the following examples are given in detail in the Supporting Information.

The class of organic–inorganic hybrid crystalline porous materials referred to as metal‐organic frameworks (MOF) attracts much attention due to their potential application in gas storage, chemical separation, drug transport, and catalysis.^35^ For their theoretical description, only a few specialized methods are available in principle, as for instance UFF, UFF4MOF,^36^ and MOF‐FF. Since the latter is only parameterized for certain metal‐organic binding motifs, UFF is the only true competitor for GFN‐FF as a general black‐box FF. However, UFF shows deficiencies in the description of conjugated systems and η‐metal‐coordinated binding motifs. For a selected set of five metal‐organic polyhedra (MOP) and MOF cut‐outs depicted in Figure 1, GFN‐FF is, to our knowledge, the only method capable of performing geometry optimizations whilst keeping the initial structure intact. Fujita et al.^37^ synthesized the largest metal‐organic Goldberg polyhedra to date through a self‐assembly reaction. The structure consists of 3888 atoms in total and is made of 46 Pd^2+^ ions coordinated square‐planar by 96 organic ligands. Charge neutrality is conserved by two BF_4_^−^ molecules per palladium ion. With GFN‐FF, the structure is optimized properly. An overlay with the crystal structure is shown in Figure 1 A. The heavy‐atom root‐mean‐square deviation (RMSD) of only 0.75 Å indicates an excellent agreement between experiment and theory. For the second‐largest MOP, shown in Figure 1 B, a similarly accurate result is obtained. Gong et al.^38^ performed a bottom‐up construction of supramolecular organic polyhedra with tetrahedral symmetries. In a non‐covalently bound three‐dimensional mesh of tetrahedra, each tetrahedral corner consists of vanadium‐oxide clusters. For the geometry optimization, the structural motif was truncated in a star‐like shape to end up at 2496 atoms in total, as shown in Figure 1 C. With an RMSD of 0.54 Å, the GFN‐FF‐optimized structure agrees very well with the experiment. A triangular channel framework constructed of Fe^III^_2_(BDP)_3_ units (BDP^2−^=1,4‐benzenedipyrazolate) was synthesized by Herm et al.^39^ A cutout of 4064 atoms, shown in Figure 1 D, was chosen and optimized by GFN‐FF, yielding an RMSD of only 0.43 Å. With 1344 atoms, the cuboctahedron Co^II^_12_L_6_, hosting two C_60_ fullerene molecules, synthesized by Rizzuto et al.^40^ and shown in Figure 1 E, is small enough to be described using SQM methodology. However, the electronic structure of twelve cobalt ions and eight negatively charged borate clusters leading to an overall molecular charge of +16 is too difficult and hence, the iterative self‐consistent‐field calculations fail to converge. On contrary, GFN‐FF is able to describe the structure with an RMSD of 1.22 Å compared to the experimental crystal structure. This somewhat larger RMSD is mainly caused by a movement of the non‐covalently bound borate clusters due to the absence of confining crystal‐packing effects in the molecular calculation.

**Figure 4:**
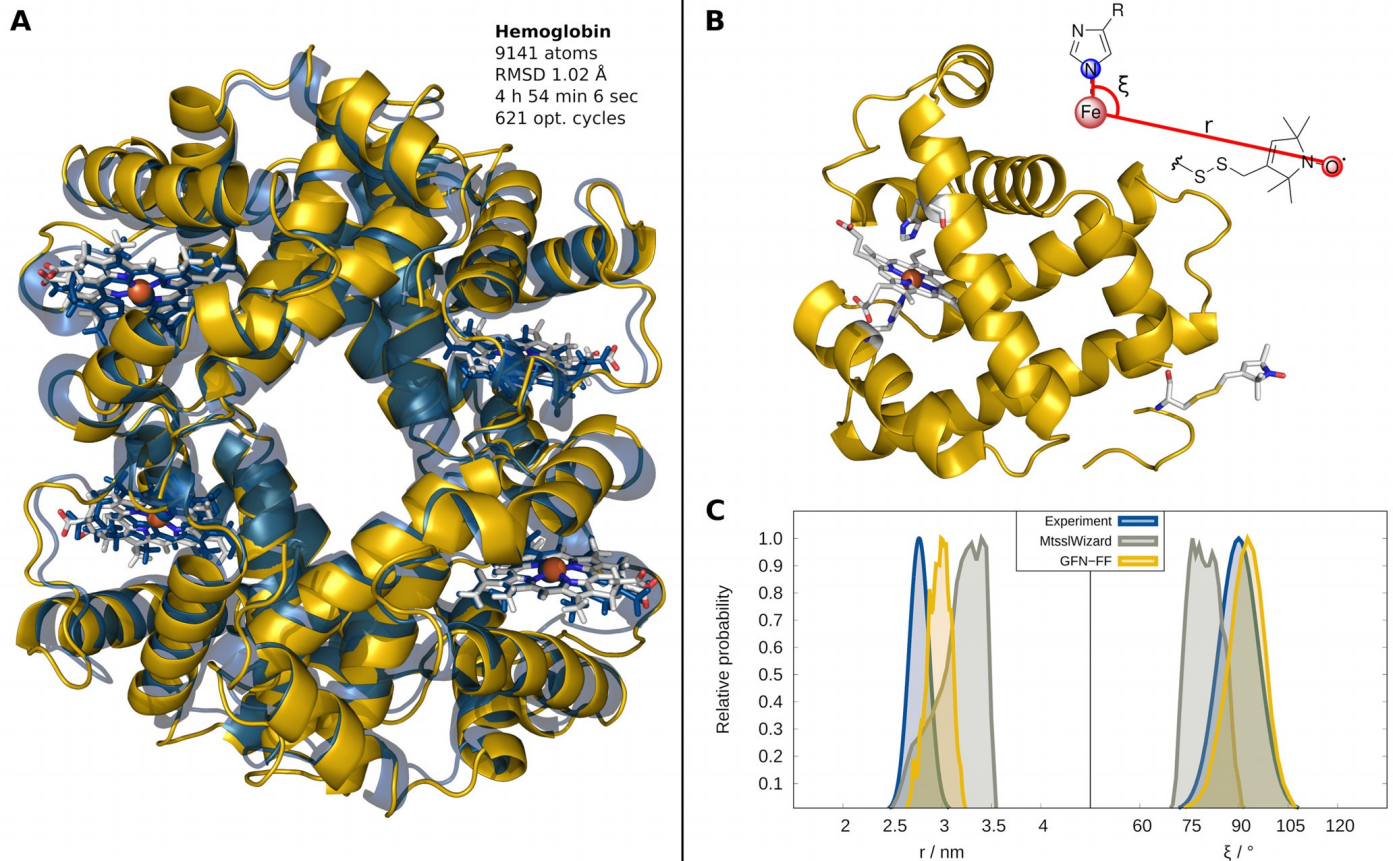
Structures and dynamics of metallo‐proteins. A) Geometry optimization of the hemoglobin structure using the GFN‐FF/GBSA(H_2_O) method. B) Myoglobin mutant Q8R1 with an open‐shell iron(III) and a nitroxide spin label covalently attached. C) Comparison of *P*(*r*) and *P*(*ξ*) to experimental EPR data and previous MtsslWizzard results.

To further show the excellent performance of GFN‐FF, the molecular structure of hemoglobin was optimized, starting from a molecular‐crystal‐structure cut‐out. The theoretical treatment is challenging here because of the co‐existence of a large bio‐organic framework (overall ≈9000 atoms) and the complicated organometallic heme groups. The overlay of the crystal structure (yellow) and the GFN‐FF‐optimized geometry (blue) of hemoglobin is show in Figure 4 A.

The calculation was conducted with an implicit generalized Born (GB) solvation model augmented by the solvent‐accessible surface area (SA). This GBSA solvation model is implemented in the *xtb* program and available for GFN‐FF. Inclusion of solvation effects is essential for the accurate modeling of bio‐macromolecules or porous materials in order to prevent structures from a “gas‐phase collapse”. Within only 5 h and 621 optimization cycles on four Intel Xeon E5‐2660 v4 @ 2.00 GHz CPUs, a stationary point on the PES was found. The heavy‐atom RMSD between the experimental and the GFN‐FF structure is only 1.02 Å, which is an excellent result for such a comparison. It shows that GFN‐FF is an efficient and technically robust FF with a physically reasonable potential that is capable of describing amino acids and metal‐containing heme groups similarly well. Due to the current occurrences, COVID‐19, the illness caused by the novel coronavirus, is in the focus of clinical research. To show the utility of GFN‐FF, a successful geometry optimization was performed on the COVID‐19 main protease in complex with an inhibitor N3 starting from the crystal structure. Again, a small RMSD between the theoretical and experimental structure of 0.95 Å is found (for details, see the Supporting Information).

One of the prime application of force‐field methods are MD simulations. From the obtained (space‐time) trajectory of the atoms, geometrical and molecular properties can be derived and can be directly compared to the experiment. Abdullin et al.^41^ conducted electronic paramagnetic resonance (EPR) measurements on a met‐myoglobin mutant (Q8R1) shown in Figure 4 B. EPR spectroscopy was used to measure the average distance *r* between a high‐spin Fe^3+^ ion and a nitroxide spin label termed MTSSL as well as the angle *ξ* formed by the iron ion, its nitrogen ligand of a histidine amino acid, and the nitroxide group. To obtain the radial distribution *P*(*r*) and angular distribution *P*(*ξ*) for Q8R1, the structure and dynamics are determined in silico by GFN‐FF and compared to experimental EPR values and previous theoretical estimates performed with MtsslWizard.^42^ MtsslWizard is a program that searches for possible MTSSL conformations that do not clash with a static model of the protein. Such a simple model is often the method of choice, since established protein force fields neither provide parameters for metals nor for the chosen nitroxide spin label. With GFN‐FF MD simulations are carried out for 1 ns at 298 K employing the GBSA(H_2_O) solvation model (see Figure 4 C). Compared to the maximum in the EPR‐measured distance distribution, GFN‐FF shows a deviation in the maximum of only 3 Å, which is within the experimental error. For the angular distribution, the difference is only 2° and hence, the GFN‐FF dynamical‐structure average is in almost perfect agreement with the experiment. This again demonstrates the accuracy of the presented FF and confirms the reliability of the calculated PES also for non‐equilibrium situations. The results with MtsslWizard (deviations for maxima of *P*(*r*) of 9 Å and 15° for *P*(*ξ*)) are clearly worse. A related study on the B1 immunoglobulin‐binding domain of a protein termed GB1 using a tailored FF for Cu‐containing metallo‐proteins appeared recently.^43^ The laborious “hand‐made” parameterization described could have been completely avoided by using GFN‐FF, which is available not only for copper but also all other transition metals.

**Figure 5:**
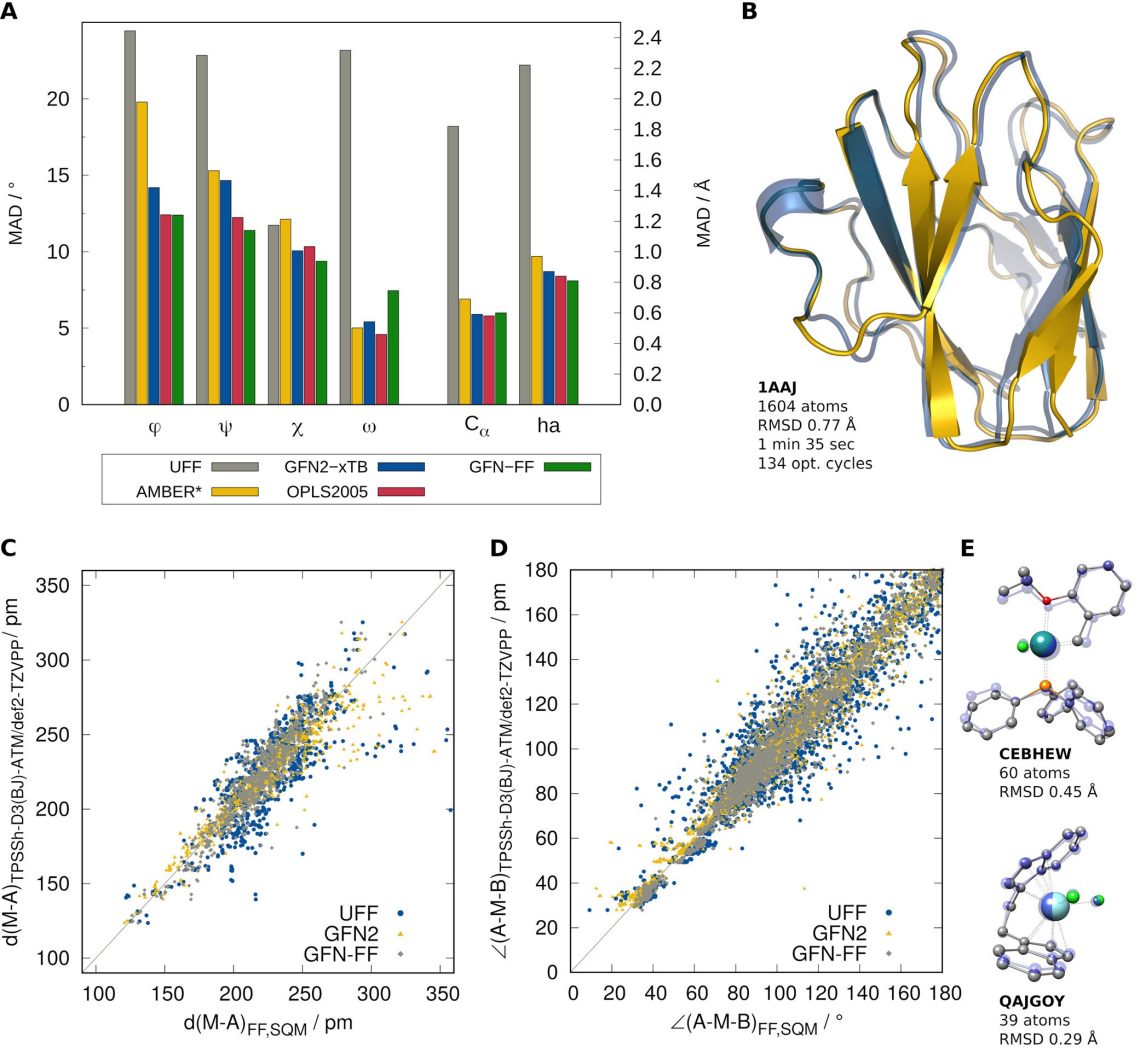
Comparison of GFN‐FF to established theoretical methods. A) Average deviations of four types of dihedral angles (in degrees) for 70 protein structures with respect to the crystal structure as well as average C_α_ and heavy‐atom RMSDs (in Å). B) Example protein structure. C), D) Performance for computed bond lengths and angles of the TMG145 benchmark set. Correlation plots for bond lengths and angles obtained with GFN2‐xTB, GFN‐FF, and UFF with reference to DFT structures. E) Example complexes from the TMG145 set.

The performance of GFN‐FF compared to other general as well as highly specialized FFs is depicted in Figure 5 for a benchmark set of 70 organic peptide and protein structures,^44^ where geometry optimizations with OPLS2005,^45^ AMBER*,^46^, ^47^ UFF, and GFN2‐xTB were conducted. Figure 5 A shows the average deviations of four dihedral angles (in degrees) and the C_α_ and heavy‐atom RMSD values in Å with respect to the X‐ray structures. A structural example is shown in Figure 5 B. The deviations of the angles *ϕ*, *ψ*, *χ*, and *ω* are soft descriptors regarding local displacements of the protein backbone and shown in detail in the Supporting Information. For the angles *ϕ*, *ψ*, and *χ*, GFN‐FF yields about the same or even better accuracy as the special‐purpose method OPLS2005 and is clearly more accurate than AMBER*. The larger deviations for *ω* indicate that the barrier for rotation around the peptide C−N bond seems to be underestimated by GFN‐FF, which is only observed for larger proteins but not for smaller peptides. The RMSD for C_α_ and heavy atoms are comparable to the much more elaborate GFN2‐xTB QM method. For a general FF that has not been specifically developed for proteins, GFN‐FF performs overall excellently on the tested protein structures. On the contrary, UFF provides large deviations for the angles *ϕ*, *ψ*, and *ω* as well as for the C_α_ and heavy‐atom RMSDs, indicating that protein structures are not well described by UFF.

For transition‐metal complexes, the quality of theoretical structures is tested on the challenging TMG145 benchmark set^48^ (for structural examples, see Figure 5 E). The performance of GFN‐FF is compared to UFF and GFN2‐xTB with reference to high‐quality DFT‐optimized structures (TPSSh‐D3(BJ)‐ATM/def2‐TZVPP). In Figure 5 C, 941 bond lengths *d*(M−A), including mainly the transition‐metal ligands, are shown. With a mean absolute deviation (MAD) of 9.7 pm, GFN‐FF performs just as good as the GFN2‐xTB QM method with a MAD of 8.3 pm. Furthermore, the reproduction of 2846 bond angles around the transition‐metal center ∡(A−M−B) is compared in Figure 5 D. Again, GFN‐FF (MAD=5.7°) performs similar to GFN2‐xTB (MAD=3.9°). For angles and bond lengths, GFN‐FF clearly outperforms UFF, which yields MAD values of 14.6 pm and 8.4°, respectively. All 145 GFN‐FF‐optimized structures fulfill the previous chemical‐correctness criteria used to identify structures that are chemically transformed, dissociated, or critically deformed during optimization, while UFF produces 75 out of 145 structures completely wrong. The performance and robustness of GFN‐FF for transition‐metal complexes is outstanding and unmatched by its direct competitor UFF.

**Figure 6:**
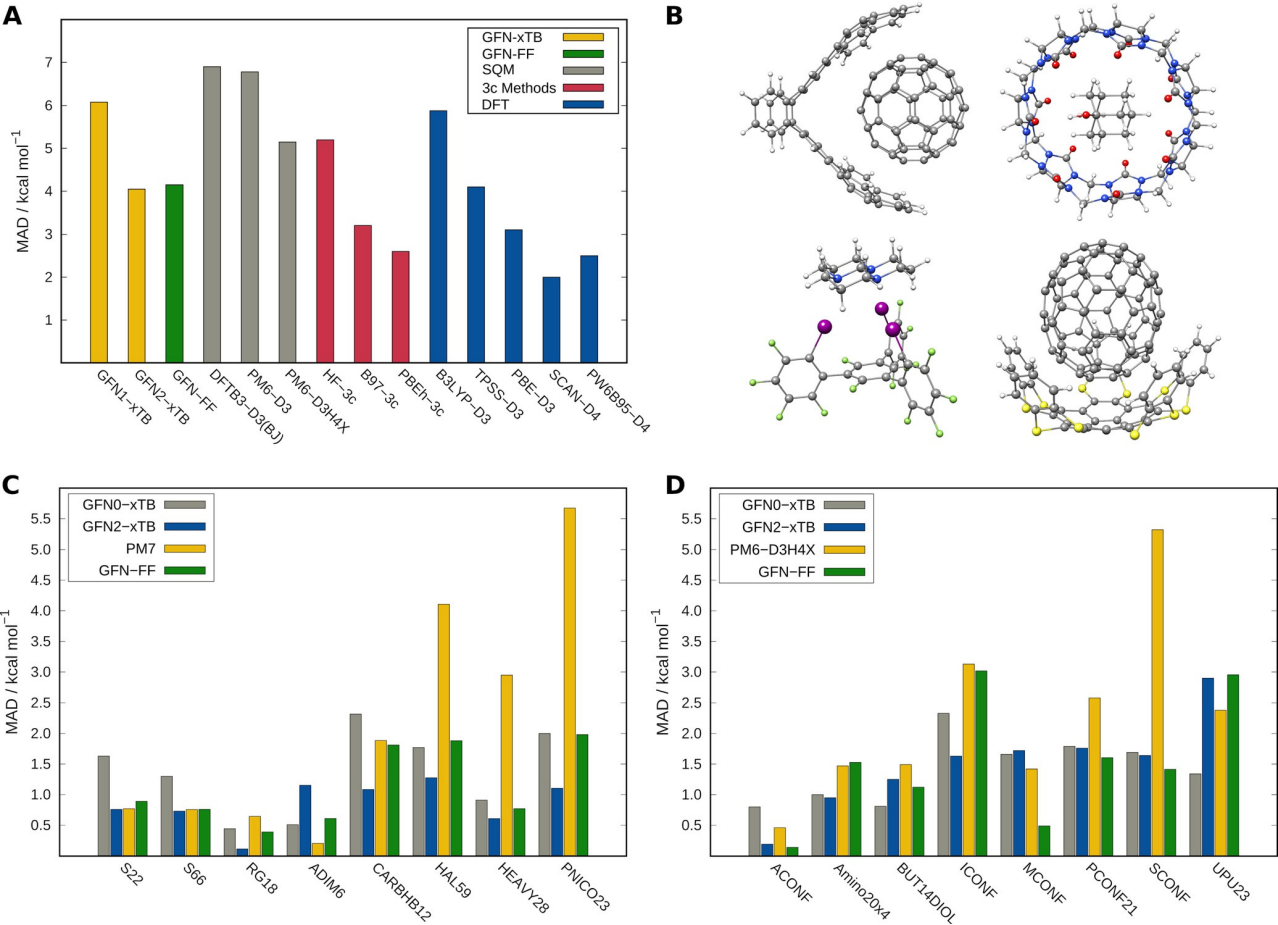
Comparing the GFN‐FF performance for established benchmark sets. A) Association energies averaged over all systems in the S30L set computed with different methods. Reference energies were obtained using the DLPNO‐CCSD(T)/CBS method. B) Four examples of structures. C) MAD values for intermolecular non‐covalent‐interaction sets. D) MAD values for several conformational benchmark sets, including the two xTB methods, GFN‐FF, PM7, and PM6‐D3H4X.

As the previous results and comparisons suggest, GFN‐FF provides almost as accurate results for equilibrium structures as sophisticated QM methods. To investigate this further for interaction energies, GFN‐FF results are compared to QM results on various interaction‐energy benchmark sets as shown in Figure 6. Intermolecular non‐covalent interactions are investigated on supramolecular host–guest systems taken from the S30L benchmark.^49^ In Figure 6 A, the performance of GFN‐FF and other QM methods is shown compared to DLPNO‐CCSD(T)/CBS reference values.^28^ The MAD of the association energy for the entire test set is given, and Figure 6 B shows four example complexes. With an overall MAD of 4.15 kcal mol^−1^, GFN‐FF outperforms most of the SQM methods and is even on par with some dispersion‐corrected DFT methods. In Figure 6 C, the performance of GFN‐FF is shown for all NCI energy subsets of the huge GMTKN55 data base.^50^ With an overall MAD of 1.13 kcal mol^−1^, the accuracy of GFN‐FF is comparable to that of GFN1‐xTB and outperforming the SQM method PM7. The GMTKN55 subsets dealing with conformational energies are shown in Figure 6 D. Again, GFN‐FF is just as good as the GFN‐xTB QM methods with an MAD of 1.53 kcal mol^−1^. Here, the excellent performance for the relative energies of alkane (ACONF) and melatonin (MCONF) conformers is noted.

## Conclusion

The development of accurate polarizable force fields is named as one of the remaining holy grails for computational chemistry.^8^ With GFN‐FF, a generic, partially polarizable force field is presented that is unique in its universality and accuracy, meeting, to a large extend, the specified requirements named in Ref. 8. It is a robust and fully automated black‐box method for the modeling and design of materials, organometallic, and biochemical systems. The performance is tested on various examples and many established benchmark sets. For biological macromolecules, it is shown that GFN‐FF is able to simulate the dynamics of a met‐myoglobin mutant and reproduces the experimental EPR‐distance measurements excellently. For a test set of 70 protein equilibrium structures, GFN‐FF performs similar or even slightly better than highly specialized protein force fields. For metal‐organic materials, GFN‐FF is, in many cases, the only currently applicable method, and for a highly complex test set of transition‐metal complexes, it exceeds the only real competitor UFF by far in terms of accuracy, robustness, and efficiency. For structures and energies, GFN‐FF is approaching the accuracy of semiempirical QM methods, in some cases reaching even DFT accuracy. The main limitation of GFN‐FF (similar to all other non‐reactive force fields) is that the input structure must be reasonably close to a “normal” chemical‐bonding situation such that the initial topology analysis works properly. If this is not the case, a few pre‐optimization steps with a GFN‐xTB QM method to adjust the covalent‐bonding network may be applied. In this work, a new quality standard is set for general force fields, providing high universality paired with almost QM accuracy at still high computational speed. An easy‐to‐use and freely available computer program implementing GFN‐FF can be downloaded for extended applications in physical and bio‐chemistry.^51^

## Conflict of interest

The authors declare no conflict of interest.

## Supporting information

As a service to our authors and readers, this journal provides supporting information supplied by the authors. Such materials are peer reviewed and may be re‐organized for online delivery, but are not copy‐edited or typeset. Technical support issues arising from supporting information (other than missing files) should be addressed to the authors.

Supplementary
