# Supplementary material for: Robust Atomistic Modeling of Materials, Organometallic, and Biochemical Systems
Source: Angew Chem Int Ed Engl. 2020 May 18;59(36):15665–73. doi: 10.1002/anie.202004239 (PMC7267649; doi:10.1002/anie.202004239)
Supplement: Supplementary file 1 — Supplementary [file ANIE-59-15665-s001.pdf]

Angewandte Chemie
Eine Zeitschrift der Gesellschaft Deutscher Chemiker

Supporting Information

Robust Atomistic Modeling of Materials, Organometallic, and Biochemical Systems

*Sebastian Spicher and Stefan Grimme**

anie_202004239_sm_miscellaneous_information.pdf

Supporting Information

Supplementary Text:

Section S1. Covalent topology

Section S2. The GFN-FF total energy

Section S3. Use of additional fragment charge information

Section S4. Potential energy curves

Section S5. Parameterization strategy

Section S6. Trouble-shooting

Materials and Methods:

Section S7. Computational details

Section S8. Benchmark data

Section S9. The description of water

Section S10. Thermostatistical corrections

Figure S1. Torsion and bend angle of the non-covalent interaction of water and formaldehyde.

Figure S2. Potential energy curves for diatomic molecules.

Figure S3. Depiction of the four dihedral angles.

Figure S4. RMSD of Q8R1 as a function of time.

Figure S5. Geometry optimization of the COVID-19 main protease in complex with an inhibitor N3.

Figure S6. Performance on the S30L benchmark set.

Figure S7. Noncovalent interaction energies from the GMTKN55.

Figure S8. Conformational energies from the GMTKN55.

Figure S9. Timings for energy and gradient computation of different GFN methods.

Figure S10. Performance of GFN-FF for the description of water.

Figure S11. Comparison of GFN-FF thermostatistical data with corresponding B97-3c DFT reference values.

Table S1. Organic protein benchmark set.

Table S2. TMG145 benchmark set.

Table S3. Association energies of the S30L benchmark set.

Table S4. Performance of different methods on the S30L benchmark.

Table S5. MAD of noncovalent interaction energies computed with GFN-FF and other semiempirical QM methods.

Table S6. MAD of conformational energies computed with GFN-FF and other semiempirical QM methods.

Table S7. Average MAD of the noncovalent and conformational energies for the GMTKN55 benchmark set.

References (1-34)

Supplementary Text

In the supporting information (SI), the theory behind GFN-FF will be explained in some detail and additional data will be provided for the results presented in the main article. The SI is organized in the following way. First the construction of the covalent topology within the force-field setup routine will be explained, followed by a detailed description of all relevant potential energy functions and the underlying parameterization strategy. Potential energy curves are shown for small diatomic molecules to highlight the dissociative nature of the force-field. Further, the computational details will be given as well as all necessary data to reproduce the results from this work. For a complete description of the technical implementation (which can not be given here due to its huge complexity), the reader is referred to the free `xtb` source code.[1]

Section S1. Covalent topology

In GFN-FF the covalent topology is coded in neighbor lists. In a first step neighbor lists are generated according to highly selective inter-atomic distance criteria, where the actual

distance between two atoms R_{AB}, is compared to a pre-computed reference value R^0_{AB} describing a common bonding distance between those two atoms in their actual environment. It is calculated according to

$$R^0_{AB} = \left(R^0_A + R^0_B + R_{sft}\right) \left(1 - c_1|\Delta EN| - c_2|\Delta EN|^2\right), \quad (1)$$

where R^0_A and R^0_B are modified CN dependent D3 damping radii and c_1 and c_2 are period-specific parameters. The parameters are fitted to reproduce PBEh-3c (a few) and B97-3c (mainly) equilibrium bond lengths using about 15–20 reference molecules per element. For the atomic electronegativity (EN), Pauling values are used. R_{sft} is an empirically determined element specific shift to the interatomic distance. For $R_{AB} < f_R(q)R^0_{AB}$ a covalent bond is assigned between atoms A and B, where $f_R(q)$ is a charge-dependent scaling function, that is unknown at this point and therefore set to unity. Depending on the number of neighbors, a hybridization state is assigned to each atom. With the information of neighboring atoms and their formal hybridization state at hand, π-conjugated fragments can be assigned with a corresponding number of π-electrons to separated parts of the entire system. Based on this initial topology, a first set of topology based electronegativity equilibrium (EEQ) charges $\mathbf{q_t}$ (see Section S2) is derived. Having atomic charges at hand the procedure is iterated once, applying $\mathbf{q_t}$ to generate the function $f_R(q)$. This results in an improved description of the topology that is propagated in a better π-fragment assignment and an improved set of topological charges within only one iteration step. Applying more then one iteration did not lead to further improvements of the results. Subsequently, ring systems are identified automatically and this information is included up to a ring size of six atoms.

The proper description of π-conjugated systems is one of the key ingredients for an accurate force-field. In GFN-FF, Hückel theory is applied to describe conjugation for the elements B, C, N, O, F, P and S. This is done in an iterative fashion, where the off-diagonal elements of the Hückel matrix depend on the density matrix P_{AB} in order to avoid over-delocalization. For more details the reader is referred to the `xtb` source code. The former π-assignment is used and for each fragment a Hückel matrix is set up. Matrix diagonalization is performed

in parallel, returning a set of eigenvalues $\epsilon_\mathbf{i}$ and eigenvectors $\mathbf{c_i}$, which are then used to construct the ground state density matrix according to

$$P_{AB} = \sum_i n_i c_{iA} c_{iB}. \tag{2}$$

The Fermi smearing technique[2] is used allow for fractional orbital occupations n_i throughout this routine. This ensures that electronically degenerate and (poly)radical situations are treated automatically. Hückel theory introduces quantum mechanics to GFN-FF and provides important bonding information as used previously in MMFF94.[3] This comes at the cost of cubic scaling with regard to the number of π-electrons in the Hückel subsystems and thus, the formal scaling of the GFN-FF setup is for large π-conjugated molecules $\mathcal{O}(N^3)$. However, due to the fact that normally only small π-fragments are taken into account and the entire setup procedure is called just once, the overall computational costs remain low. Nevertheless, the program has been successfully tested for molecule adsorption problems on huge graphene flakes with about 5000 atoms.

With a complete topology assignment and knowledge about atomic charges, ring- and π-systems, in combination with the set of element specific fitted parameters, all force constants (FC) and relevant equilibrium values for bond, bend and torsion potentials can be derived. Along with the other energy terms as discussed below, the total energy E and its analytical gradient g are calculated. In GFN-FF the entire setup routine and calculation of energy and gradient, as formulated above, is executed fully automatic. To highlight the simplicity of how to use GFN-FF for example to conduct a geometry optimization, the command line input for the `xtb` program is given explicitly below,

```
xtb input.xyz --gfnff --opt > xtb.out
```

where input.xyz contains the molecular starting geometry in a standard xyz format. To the authors knowledge and practical experience, GFN-FF as implemented in `xtb` is by far one of the most user friendliest force-fields available.

Section S2. The GFN-FF total energy

The total GFN-FF energy expression is given by

$$E_{GFN-FF} = E_{cov} + E_{NCI}, \tag{3}$$

where E_{cov} refers to the bonded force-field energy often termed strain energy. E_{NCI} describes the intra- and inter-molecular noncovalent interactions, also called non-bonded terms. In the following atomic units are used throughout. The covalent E_{cov} part contains a sum of covalently bonded (bond), bending angle (bend), torsion angle (tors), repulsion (rep) and three-body (abc) dependent terms according to

$$E_{intra} = E_{bond} + E_{bend} + E_{tors} + E_{rep}^{bond} + E_{abc}^{bond}, \tag{4}$$

where E_{bond} is the potential energy for stretching a bond between two atoms A and B. E_{bend} is the potential energy required for bending an angle formed by three atoms A-B-C, where a covalent bond exists between A and B, as well as between B and C. E_{tors} describes the part of the potential energy associated with the rotation around a B-C bond in a four-atom sequence A-B-C-D, where A-B, B-C and C-D are bonded. E_{rep}^{bond} is the repulsion energy for all bonded atom pairs.

The noncovalent interaction energy E_{NCI} is composed of the isotropic electrostatic (IES) energy, dispersion (disp) and Pauli repulsion (rep) energy, as well as a hydrogen bond (HB) and halogen bond (XB) terms:

$$E_{NCI} = E_{IES} + E_{disp} + E_{HB} + E_{XB} + E_{rep}^{NCI}. \tag{5}$$

Bond term

For a covalent bond between two atoms A and B the energy E_{bond} is calculated by a newly developed Gaussian type function

$$E_{bond} = \sum_{bonds} -k_{str} \cdot exp\left[-\eta_{bnd}\left(1 + k_{EN}|\Delta EN(AB)|^2\right) \cdot \left(R_{AB} - R_{AB}^0\right)^2\right], \tag{6}$$

which allows smooth dissociation of the AB bond into the separated atoms. This is a reversible process allowing broken bonds to reform. However the formation of new bonds is not possible. In Eq. 6, R_{AB} is the actual interatomic distance, R^0_{AB} is the equilibrium value (Eq. 1) and η_{bond} and k_{EN} are global fitting parameters. ΔEN is the difference between the EN of the elements $\Delta EN = EN_A - EN_B$. k_{str} is the bonding force constant, which is determined as a function of the coordination number (f_{CN}), atomic charges (f_{qq}), π-bond character (f_π), element type (main group metal or heavy element f_{hvy}) and whether the atoms are part of a ring system f_{rng})

$$k_{str}(AB) = f_{CN} \cdot f_{qq} \cdot f_\pi \cdot f_{hvy} \cdot f_{rng} \cdot k_b(A)k_b(B). \tag{7}$$

k_b represent element specific bond parameters determined from the fit to reference data. In general, an element specific parameter that represent a function of global parameter and atomic properties will be termed with an f. In terms of FCs, element specific parameter that where determined by a fit to reference data will be termed with a k. If a covalent bond is of OH or NH type and and the O/N atoms are involved in hydrogen bonding, the exponent in Eq. 6 is multiplied by the function f_{CN_H},

$$f_{CN_H} = (1.0 - 0.1\,CN_H) \tag{8}$$

where CN_H is the modified D3 coordination number (mCN) of the hydrogen atom, that is part of the bond and also the central atom within a hydrogen bond. The mCN is discussed in the dispersion part. For bonds involved in hydrogen bonding motifs, this modification of Eq. 6 models the population of the anti-bonding σ^* orbital by the hydrogen bond acceptor atom and the resulting weakening/elongation of the respective bond. As a result, GFN-FF reproduces the splitting of, e.g., the OH stretching vibrational band in the spectrum of the water dimer.

Bending term

For the bending term involving three atoms A-B-C, where A is the central atom, the following

energy expression is employed for angles θ^{abc},

$$E_{bend} = \sum_{bend} f_{dmp} k_{bnd} \begin{cases} \left(\theta^{abc} - \theta_0^{abc}\right)^2 & \theta_0 \approx \pi \\ \left(cos(\theta^{abc}) - cos(\theta_0^{abc})\right)^2 & \text{else} \end{cases} \tag{9}$$

where a case distinction is made between equilibrium angles close to linearity ($\theta_0 \approx \pi$) and all other cases, which apply a double-minimum function that allows inversion. The FCs k_a represent element specific parameters. Here, k_{bnd} is the bending FC, which is determined as a function of the atomic charges (f_{qq}) also including a correction for small angles f_{sml} and metals as the central atom of the angle f_{mtl}

$$k_{bnd}(ABC) = f_{qq} \cdot f_{sml} \cdot f_{mtl} \cdot k_a(A) k_a(B) k_a(C). \tag{10}$$

k_a represent element specific angular bending parameters determined from the fit to reference data. $f_{dmp} = f_{dmp}(AB) f_{dmp}(AC)$ is the product of the distance dependent damping functions (modified from Ref.[4]) for AB and AC that are given by,

$$f_{dmp}(AB, R) = \frac{1}{1 + k_{dmp} \left(\frac{R_{AB}}{R_{AB}^{cov}}\right)^4} \tag{11}$$

with the covalent distance for the pair $R_{AB}^{cov} = R_A^{cov} + R_B^{cov}$ as the sum of covalent atomic radii[5] and a global parameter k_{dmp}, chosen such that the potential vanishes approximately at twice the covalent distance. This damping of bending as well as torsional terms (see below) for long distances allows proper dissociation of the molecule into atoms.

Torsion term

The energy expression E_{tors} for a rotation around a bond AB with atom C connected to A and atom D to B is given by,

$$E_{tors} = \sum_{torsion} f_{dmp} k_{tor} \left[1 + cos\left(n(\psi - \psi_0) + \pi\right)\right] \tag{12}$$

where f_{dmp} is the triple damping product in analogy with the bending term (for CA, AB, BD) and ψ_0 is the equilibrium torsion angle. The appropriate multiplicity number n describes a rotation that is periodic by 360° for $n = 1$, the $n = 2$ term is periodic by 180°, the $n = 3$

term is periodic by 120° and so on. k_{tor} is the torsion force constant, which determines the size of the rotational barrier around the A-B bond. It is determined as a function of the σ- (f_σ) and π- (f_π) character of the A-B bond and of the atomic partial charges (f_{qq})

$$k_{tor} = f_\sigma \cdot f_\pi \cdot f_{qq} \cdot k_t(AB)k_t(CD). \tag{13}$$

k_t represent element specific torsion parameters determined from the fit to reference data. Out-of-plane or improper torsion terms are constructed in the same manner, taking into account all three-fold coordinated sp^2-hybridized atoms, π-atoms and additionally three-coordinated nitrogen atoms. This choice yields physically correct double-minimum potentials and proper inversion barriers. For out-of-plane and improper torsion a different force constant k'_{tor} is applied.

Repulsion term

Unlike other force-fields, where harmonic potentials, generalizations of Lennard-Jones[4] or Morse-type potentials are applied to describe bond stretching, in GFN-FF bond elongation and compression are considered separately. As a novelty in GFN-FF, repulsive forces arising upon bond compression are treated in analogy to classical nuclear repulsion terms as in Ref.[6]

$$E_{rep}^{bond} = \sum_{A,B} \eta_{rep}^{bond} \frac{Z_A^{eff} Z_B^{eff}}{R_{AB}} exp\left(-\sqrt{\alpha_A \alpha_B R_{AB}^3}\right). \tag{14}$$

Here Z_A^{eff} and Z_B^{eff} are effective nuclear valence charges of atoms A and B, which, like α_A and α_B are element specific parameters. η_{rep}^{bond} is a global scaling parameter of the repulsion energy especially for covalently bond atoms.

Bonded three-body term

For the description of three-body effects the well-known ATM term[7,8] taken from the theory of dispersion forces is adopted here for the covalent regime. Therefore, the atoms A-B and B-C must be covalently connected. Such a term has never been used before in a force-field. In GFN-FF, the following expression is used to describe a correction to the interaction between

three covalently bond atoms A, B and C

$$E_{abc}^{bond} = \sum_{ABC} C_{abc} \frac{(3\, cos\theta_a\, cos\theta_b\, cos\theta_c + 1)}{(R_{AB}R_{AC}R_{BC})^3}. \tag{15}$$

Here, θ_a, θ_b and θ_c are the internal angles of the triangle formed by R_{AB}, R_{BC} and R_{AC} respectively. In comparison to the description of three-body effects in the context of dispersion interaction, the triple-dipole constant C_9^{ABC} is replaced by the triple-nuclear constant C_{abc} given by

$$C_{abc} = f_{q,a} f_{q,b} f_{q,c}\, \eta_{abc}\, (Z_A Z_B Z_C)^{1/3}, \tag{16}$$

where f_q are linear functions of the respective atomic charge. η_{abc} is a global parameter and Z is the scaled nuclear charge. E_{abc}^{bond} may be considered as a non-additive three-body correction to covalent binding which appears in QM methods as many center nuclear-electron attraction and electron-electron repulsion integrals, respectively.

Electrostatic energy

For the description of isotropic electrostatic (IES) energy, a classical charge model based on electronegativity equilibration (EEQ) of Gaussian type model charge densities is used, as previously applied in the DFT-D4 scheme.[9] This EEQ model is also capable of describing the total charge of charged species as the sum of atomic charges and allows charge distribution on the whole system. The IES energy expression, which is minimized variationally, is given by

$$E_{IES} = \sum_{A} \left[\chi_A q_A + \frac{1}{2} \left(J_{AA} + \frac{2\gamma_{AA}}{\sqrt{\pi}} \right) q_A^2 + \sum_{A>B} q_A q_B \frac{erf(\gamma_{AB} R_{AB})}{R_{AB}} \right], \tag{17}$$

or within matrix notation as

$$E_{IES} = \mathbf{q}^{\mathbf{T}} \left(\frac{1}{2} \mathbb{A} \mathbf{q} - \mathbf{X} \right), \tag{18}$$

where elements of the $\mathbf{X}$ vector and elements of the $\mathbb{A}$ matrix are given by

$$X_A = \Omega_A - EN_A, \tag{19}$$

with

$$A_{AB} = \begin{cases} J_{AA} + \frac{2\gamma_{AA}}{\sqrt{\pi}} & \text{for A = B,} \\ \frac{erf(\gamma_{AB} R_{AB})}{R_{AB}} & \text{otherwise.} \end{cases} \tag{20}$$

Here, $\gamma_{AB} = \frac{1}{\sqrt{a_A^2 + a_B^2}}$ with the atomic radii a_A and J_{AA} represents element dependent atomic hardness. The modified electronegativity X_A is computed from the fitted atomic electonegativities EN_A and a scaled logarithmic coordination number (CN) Ω_A

$$\Omega_A = \kappa_A \sqrt{log\left(\frac{1 + exp(CN_{max})}{1 + exp(CN_{max} - mCN_A)}\right)}, \tag{21}$$

where κ_A is an element dependent scaling factor, CN_{max} the maximum CN and mCN_A the modified D3 CN

$$mCN_A = \frac{1}{2} \sum_{B \neq A} \left[1 + erf\left(-7.5\left(\frac{R_{AB}}{R_{AB}^{cov}} - 1\right)\right)\right]. \tag{22}$$

The logarithmic function applied has the following properties. For small values it is identical to the modified CN in Eq. 22 but for larger values it approaches asymptotically the highest chemically reasonable value for a CN in the molecular case (CN_{max}). This behavior of Ω_A prevents the modified electronegativity X_A to adopt nonphysical values in highly coordinated systems. In molecular systems the atomic charges have to sum up to the total charge of the system $\sum_A q_A = q_{tot}$. Adding this constrain in terms of Lagrange multipliers to Eq. 18 leads to the modified linear system of equations

$$\begin{pmatrix} \mathbb{A} & \mathbf{1} \\ \mathbf{1^T} & 0 \end{pmatrix} \begin{pmatrix} \mathbf{q} \\ \lambda \end{pmatrix} = \begin{pmatrix} \mathbf{X} \\ q_{tot} \end{pmatrix}. \tag{23}$$

Solving this set of linear equations leads to the geometry dependent charges q_A. This classical charge model introduces four further empirical parameters (J_{AA} , a_a , EN_A and κ_A) per element to GFN-FF. The formal scaling of the procedure is $\mathcal{O}(N^2)$ with the number of atoms N and therefore GFN-FF scales with this same order of magnitude. The analytical derivative of the partial atomic charges with respect to nuclear displacements $\frac{\partial \mathbf{q}}{\partial R_i}$ scales cubic $\mathcal{O}(N^3)$ and thus it is impractical for a fast and general force-field. Thus, in GFN-FF a second set of purely topology dependent charges q_t is introduced as mentioned already above in the section on the setup procedure. The Floyd–Warshall algorithm[10] is employed to determine the shortest covalent path between all pairs of atoms. Replacing the interatomic distance R_{AB} in Eq. 17 by the sum of all covalent radii that lie on the shortest path between atom A

and B

$$R_{AB}^{topo} = \sum_{i}^{A \rightarrow B} \eta_{topo} \, R_i^{\prime cov}, \tag{24}$$

leads to a geometry independent but purely topology based atomic partial charges. Here, η_{topo} is a global scaling parameter and the sum of covalent radii $R_{AB}^{\prime cov} = R_A^{cov} + R_B^{cov}$.[11] This topological approach eliminates the costly analytical derivative of these charges with respect to nuclear displacements but still includes the environmental effects of neighboring atoms. In addition to the dependency of the charges on the system geometry in the EEQ model, the topology based GFN-FF charges (which are used to derive various potential energy terms) take further polarization effects into account. GFN-FF is thus a partially polarizable but still efficient force-field with environment dependent atomic charges.

Dispersion energy

In GFN-FF long-range dispersion is treated by applying a modified version of the DFT-D4 scheme. Here, in analogy to the well established D3 scheme,[12,13] the pair–wise dipole-dipole dispersion coefficients are calculated from a Casimir–Polder integration over atomic dynamic polarizabilities $\alpha(i\omega)$.

$$C_6^{AB,D3} = \frac{3}{\pi} \int_0^{\infty} d\omega \, \alpha^A(i\omega) \alpha^B(i\omega). \tag{25}$$

The resulting C_6^{D3} coefficients are multiplied by a charge-scaling function ζ

$$C_6^{AB} = \zeta^A \zeta^B \, C_6^{AB,D3}, \tag{26}$$

with the atom specific charge function ζ^A defined as

$$\zeta^A \left(z^A, z^{A,ref} \right) = exp \left[\beta_1 \left\{ 1 - exp \left[\gamma^A \left(1 - \frac{z^{A,ref}}{z^A} \right) \right] \right\} \right], \tag{27}$$

where the chemical hardness γ_A is taken from Ref.[14] and determines as an element specific parameter the steepness of the scaling function while β_1 is a global parameter. The effective nuclear charge z^A is defined as the sum of the nuclear charge of atom A and the topological atomic partial charges q_t^A

$$z^A = Z^A + q_t^a. \tag{28}$$

$z^{A,ref}$ are effective nuclear charges for element specific reference systems employing modified nuclear charges for all elements beyond krypton. In contrast to the D4 model, where atomic reference polarizabilities $\alpha(i\omega)$ are scaled by ζ, in GFN-FF the charge scaling is applied to pair-wise C_6^{AB} coefficients. C_8^{AB} coefficients are computed recursively from the C_6^{AB} coefficients. The dispersion energy is calculated according to

$$E_{disp}^{(6,8)} = -\sum_{AB} \sum_{n=6,8} s_n \frac{C_{(n)}^{AB}}{R_{AB}^{(n)}} f_{damp}^{(n)} \left(R_{AB} \right), \tag{29}$$

where s_n scales the individual multipolar contributions and $f_{damp}^{(n)}$ denotes the rational Becke-Johnson (BJ) damping function.[15] Treating only two-body dispersion contributions in GFN-FF leads to a scaling of $\mathcal{O}(N^2)$ for the energy and gradient computation.

Hydrogen bond correction

For atom pairs involving the electronegative elements C, N, O, F, Si, P, S, Cl, As, Se, Br, Sb, Te and I, an additional hydrogen bonding correction is applied when as a third atom hydrogen is involved. A case distinction is made between a classical hydrogen bonding motif, where the hydrogen atom is covalently bound to the donor atom A and noncovalently interacting with a hydrogen acceptor atom B. In the second case, the H atom is noncovalently interacting with both, the donor and the acceptor atom (i.e., it is covalently bonded neither to A nor to B). The total hydrogen bonding energy is given by

$$E_{HB} = E_{HB}^{A-H\cdots B} + E_{HB}^{A\cdots H\cdots B}. \tag{30}$$

Here, the sum is taken over specific atom triples AHB (donor-hydrogen-acceptor) as

$$E_{HB}^{A-H\cdots B} = -\sum_{AHB} f_{dmp}^{srt} f_{dmp}^{lng} \Upsilon_{dmp}^{out} \chi_{AHB}^{\alpha} \left(\frac{\omega_{AB}}{R_{AB}^3} + \frac{\omega_{BH}}{R_{BH}^3} \right), \tag{31}$$

where ω_{AB} and ω_{BH} are global parameters that determine the weighting of the distance dependency between donor-acceptor (R_{AB}) and acceptor-hydrogen (R_{BH}). The dependence on the inverse third power of the distance is chosen due to the strong polarization and dipole-dipole character of hydrogen bonding which differs from the electrostatic Coulomb interaction. To smoothly interpolate between donor/acceptor character, the interaction strength is

modified to ensure that the hydrogen bonding correction vanishes for weak donor basicity and weak acceptor acidity character

$$\chi^{\alpha}_{AHB} = c_a^A \rho_q^A \, c_b^B \rho_q^B \, \rho_q^H \, \eta_{hb}. \tag{32}$$

Here η_{hb} is a global scaling parameter and c_a^A and c_b^B are global fitting parameters describing the acidity of atom A and the basicity of atom B. Charge dependencies are included for atoms A, B and H by employing the charge functions ρ_q, which are based on the topology dependent charges q_t and the global parameters k_{q1} and k_{q1}

$$\rho_q^{A,B} = \frac{exp\left(-k_{q1}\, q_t^{A,B}\right)}{exp\left(-k_{q1}\, q_t^{A,B}\right) + k_{q2}}, \tag{33}$$

and

$$\rho_q^H = \frac{exp\left(k_{q1}\, q_t^H\right)}{exp\left(k_{q1}\, q_t^H\right) + k_{q2}}. \tag{34}$$

Here, f^{srt}_{dmp} is a damping function reducing the H-bond contribution to zero for short distances between donor and acceptor

$$f^{srt}_{dmp} = \frac{1}{1 + \left(\frac{\eta_{srt} R'^{cov}_{AB}}{R^2_{AB}}\right)^{\gamma_{srt}}}, \tag{35}$$

where η_{srt} and γ_{srt} are global parameters and $R'^{cov}_{AB} = R'^{cov}_A + R'^{cov}_B$. For long distances between donor and acceptor a corresponding damping function f^{lng}_{damp} is applied with η_{srt} and γ_{srt} as global parameters.

$$f^{lng}_{dmp} = \frac{1}{1 + \left(\frac{R^2_{AB}}{\eta_{lng}}\right)^{\gamma_{lng}}}, \tag{36}$$

The strength of a hydrogen bond correlates with the angle formed by the three atoms AHB involved. In order to introduce an angular dependency the out-of-line damping function Υ^{out}_{dmp} is introduced

$$\Upsilon^{out}_{dmp} = f^{out}_H \prod_i^{\Delta_B} f^{out}_i. \tag{37}$$

Here, the first out-of-line term f^{out}_H is applied so that the hydrogen bond contribution vanishes for nonlinear arrangements

$$f^{out}_H = \frac{2}{1 + exp\left[\frac{\eta^H_{out}}{R'^{cov}_A + R'^{cov}_B}\left(\frac{R_{AH}+R_{BH}}{R_{AB}}\right) - 1\right]}, \tag{38}$$

where η_{out}^{H} is a global parameter.

For the correct description of hydrogen bonding motifs, knowledge about the presence and location of paired electrons in lone-pair orbitals (LP) is crucial. Because of the absence of information on electrons and occupied orbitals in a force-field, this dependence nontrivial to realize. GFN-FF addresses the problem of how to locate the LP by a simple geometrical model. The LP is assumed to be located in maximum distance to all covalently bound neighbors of the hydrogen accepting atom, obeying the chemical VSEPR principle. The second term in Eq. 37 is the product of damping functions f_i^{out} over the number of covalently bound neighbors of the hydrogen acceptor Δ_B

$$f_i^{out} = \frac{2}{1 + exp\left[-\frac{\eta_{out}^{\Delta}}{R_A^{cov}+R_B^{cov}}\left(\frac{R_{Ai}+R_{Bi}}{R_{AB}}\right) - 1,\right]} - 1 \tag{39}$$

where η_{out}^{Δ} is a global parameter. This damping function can be regarded as the inverse of f_H^{out} and it is applied so that the hydrogen bond contribution vanishes for linear arrangements of the neighboring atoms of the acceptor atom in between the donor-acceptor bond. To the authors knowledge, this is the first attempt to introduce LPs to force-fields via an exclusion principle over neighbor lists.

For carbonyl- and nitro-groups involved as a lone-pair donor within a hydrogen bond, a torsion and bending potential is applied for the non-covalent interaction between donor and acceptor. The hydrogen bond potential is modified by,

$$E_{HB}^{A-H\cdots B} = -\sum_{AHB} f_{dmp}^{srt} f_{dmp}^{lng} \Upsilon_{dmp}^{out} \chi_{AHB}^{\alpha} \left(\frac{\omega_{AB}}{R_{AB}^3} + \frac{\omega_{BH}}{R_{BH}^3}\right) f_{tors}(\psi)\, f_{bend}(\theta), \tag{40}$$

where $f_{tors}(\psi)$ is a torsion function and $f_{bend}(\theta)$ a bending function, derived from the potentials used for covalent bending and torsion, respectively, as described above. The angles ψ and θ are defined in Figure S1. By this refinement of the HB potential, the position of the hydrogen atom is favored within the plane of the carbonyl group at an angle θ of 120° or 240° respectively, which are the positions of the lone-pairs at the carbonyl-oxygen atom.

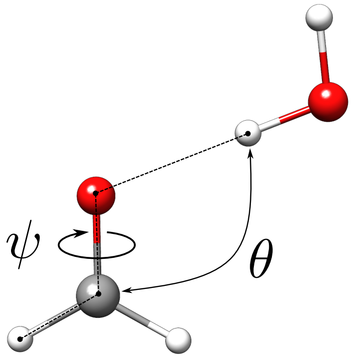

Figure S1. Torsion and bend angle of the non-covalent interaction of water and formaldehyde. Bending and torsion potentials are chosen to place the hydrogen atom of the H-bond in the carbonyl plane at an angle of 120° or 240°.

The H-bond term for the second case is given by a sum over specific atom triples AHB (donor/acceptor-hydrogen-donor/acceptor) as

$$E_{HB}^{A\cdots H\cdots B} = -\sum_{AHB} f_{dmp}^{srt} f_{dmp}^{lng} f_{H}^{out} \frac{\chi_{AHB}^{\beta}}{R_{AB}^{3}}. \tag{41}$$

Here the same short- and long-range damping functions are used as before. For the out-of-line damping only the linearity relation regarding the hydrogen atom is applied and neighbors are not further included. For symmetric H-bonds the interaction strength is modified by

$$\chi_{AHB}^{\beta} = \frac{c_a^A R_{BH}^4 + c_a^B R_{AH}^4}{R_{AH}^4 + R_{BH}^4} \cdot \frac{c_b^A \rho_q^A R_{BH}^4 + c_b^B \rho_q^B R_{AH}^4}{R_{AH}^4 + R_{BH}^4} \cdot q_t^H, \tag{42}$$

where the first term is a measure of the acidity of hydrogen donor and acceptor and the second term the corresponding basicity scaled by the topological charge of the hydrogen atom. Again, for weak donor basicity and acceptor acidity character the hydrogen bonding correction vanishes.

Halogen bond correction

The electron acceptor property of the halogens is based on the so-called sigma hole model.[16] To describe this effect in GFN-FF, a similar potential as for the hydrogen bond correction is used for the halogen bonding situation D-X-Y where atom D is any donor atom, X is the halogen or other sigma-hole containing elements[17] (P, S, Cl, As, Se, Br, Sb, Te and I) and Y

can be any acceptor atom from group 15–17. The interaction is treated similar to hydrogen bonding as a sum over all atom triples DXY

$$E_{XB} = -\sum_{DXY} f_{dmp}^{srt} f_{dmp}^{lng} f_X^{out} \frac{\chi_{DXY}^{\alpha}}{R_{XY}^3}, \quad (43)$$

where again short- and long-range damping functions are applied and f_X^{out} is the out-of-line damping function for atom X. The interaction strength χ_{DXY}^{α} in this version depends only on the halogen atom and its acceptor

$$\chi_{DXY}^{\alpha} = c_b^Y \rho_q^Y c_b^X \rho_q^X, \quad (44)$$

where c_b are again the corresponding global basicity parameters and ρ_q the charge dependent scaling functions.

Section S3. Use of additional fragment charge information

In GFN-FF the computed atomic charges from the EEQ model may be improved by constrains if additional information about the charge distribution in the system is known. There are three different ways to incorporate this information. If the system consists of more than one NCI fragment, the charges per fragment can be written by the user into a specific file (named `.CHRG`) and will be constrained accordingly in the EEQ model, thus preventing artificial charge transfer between the NCI fragments. If a GFN-xTB calculation is performed in advance, the written file "charges" is read by the program and the corresponding QM charges are used to constrain the values on the molecular fragments. The last option is useful especially for biological systems. If a "pdb" file is provided with known charges on specific residues, the `xtb` program reads this information, determines the overall charge of the system automatically and applies this charge constrain per residue, again preventing (inter-residue) charge transfer.

Section S4. Potential energy curves

GFN-FF is a dissociative force-field allowing cleavage of all covalent bonds that are assigned by the initial topological procedure. Forming new covalent bonds after the setup stage (i.e., a

reactive force-field) is not possible. Figure S2 shows the computed potential energy curves for five diatomic molecules and includes reference dissociation energies taken from Ref.[18] The asymptotic correct value is indicated by dashed lines in the same color as the corresponding GFN-FF result. For a broad range of bonds with different polarity and bond orders, GFN-FF is able to describe the dissociation qualitatively correct. No attempt in the parameter fitting procedure was made to improve the description of atomization energies, i.e., this test can be seen as a difficult cross check of the covalent part of the GFN-FF potential.

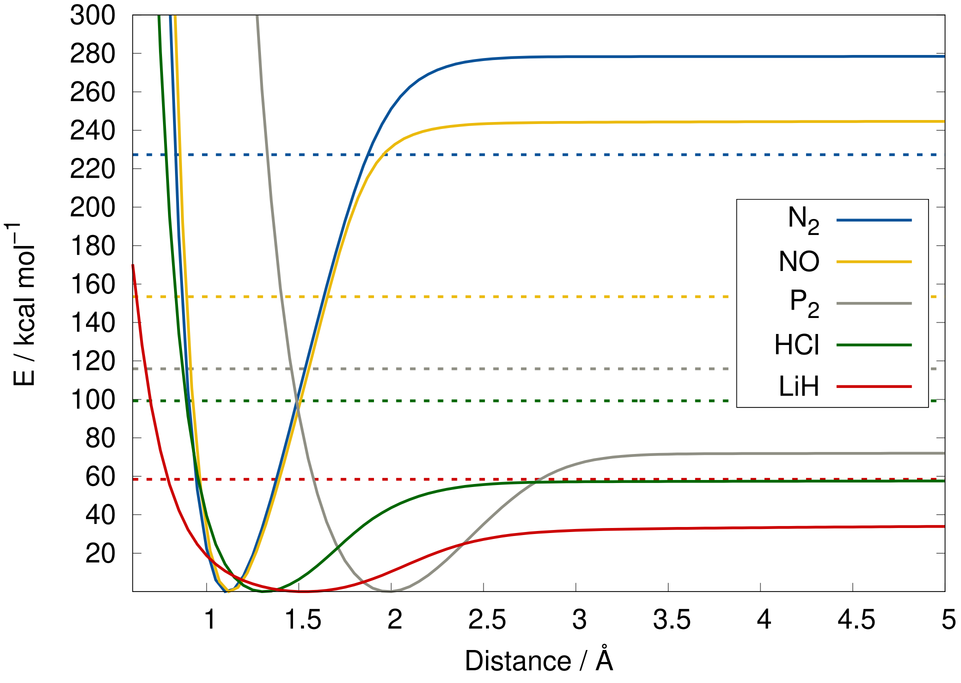

Figure S2. Potential energy curves for diatomic molecules. Computed GFN-FF potential energy curves and reference dissociation energies (asymptotic value indicated by the dashed line) for five diatomic molecules.

Section S5. Parameterization strategy

The fitting procedure is technically done with the Levenberg–Marquardt[19,20] algorithm. Applying this method, global and the element-specific parameters are determined by minimizing the root-mean-square deviation (RMSD) between reference (PBEh-3c[21] and B97-3c[22]) and GFN-FF computed data. The reference data consist of equilibrium and distorted geometries for force matching, harmonic frequencies and NCI energies of subsets from the GMTKN55

data base. The reference structure set is an extended and refined version of the GFN2-xTB training set, owing to the fact that larger systems can be treated by the force-field. Special attention is payed towards the correct description of transition-metal (TM) complexes by the inclusion of highly coordinated TM systems. Furthermore, the general fitting strategy is identical, meaning the global parameters have been determined along with the element specific parameters for the elements H, C, N and O first and the parameters for all other elements were fitted while keeping the existing (HCNO)-parameters fixed. The element specific parameters for the lanthanoids are linearly interpolated between the elements La and Lu by means of the nuclear charge Z. Due to the amount of parameters, they will not be listed in the SI. However, the `xtb` code is open source and parameters can be looked up in the code provided on our github website.[1]

Section S6. Trouble-shooting

The implementation of GFN-FF was tested to the best of our ability for very many different chemical systems. However, incorrect assignments of bond topology leading to bad results may still occur in particular for unusual element combinations. Therefore we refer to our github website,[1] where a detailed manual of how to use GFN-FF in `xtb` can be found. This website also offers the possibility to open up issues and ask questions directly. Since it is impossible for us to test every possible molecular system, in some cases GFN-FF might fail. If such a case is observed we are pleased about user feedback and we will try correct it. Updates of the program in this respect as well as other revisions will always be announced on this website. This procedure worked already well informally during development of the program/method thanks to many beta-users world-wide.

Materials and Methods

In this section, all the necessary information is given to reproduce the data in this work. The computational methods and software packages used are listed as well as all the data necessary to construct the graphics. For the given statistics, the following abbreviations are used throughout: mean deviation (MD), mean absolute deviation (MAD), standard deviation (SD) and root mean square deviation (RMSD).

Section S7. Computational details

Detailed comparisons between GFN1-xTB and GFN2-xTB with other semiempirical methods have been conducted in the respective original method publications.[6,23] Therefore we will compare the performance of GFN-FF to its predecessors and results for other semiempirical calculations, such as DFTB3-D3(BJ),[24] PM6-D3H4X[25] and PM7.[26] All GFN calculations have been performed with our standalone program termed `xtb` (version 6.3). PMx calculations have been performed with MOPAC2016 (version 18.151).[27] DFT and coupled cluster calculations have been conducted with the TURBOMOLE.7.3.1[28,29] and ORCA.4.1.1[30] program packages. Computational data for each benchmark set was taken from the respective publication.

For the geometry optimizations shown in Figure 1 and 2 in the main text, an implicit GBSA solvation model is used throughout. For all optimizations in Figure 1 convergence thresholds of $E_{conv} = 5 \cdot 10^{-6} E_h$ are employed. For the optimizations of all protein structures this value is changed to $E_{conv} = 5 \cdot 10^{-5} E_h$. MD simulations with GFN-FF are carried out for 1 ns at 298 K employing the implicit GBSA(H_2O) solvation model. A time step of 4 fs (at an increased hydrogen mass of 4 amu) and equilibration phase of 200 ps is chosen.

Section S8. Benchmark data

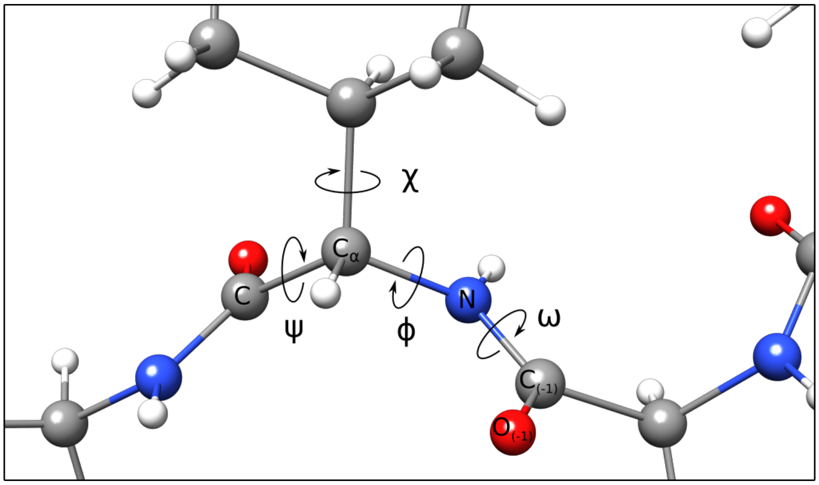

Figure S3. Depiction of the four dihedral angles. The angles ϕ, ψ and ω discussed in this work are backbone angles, whereas χ is the first side chain angle.

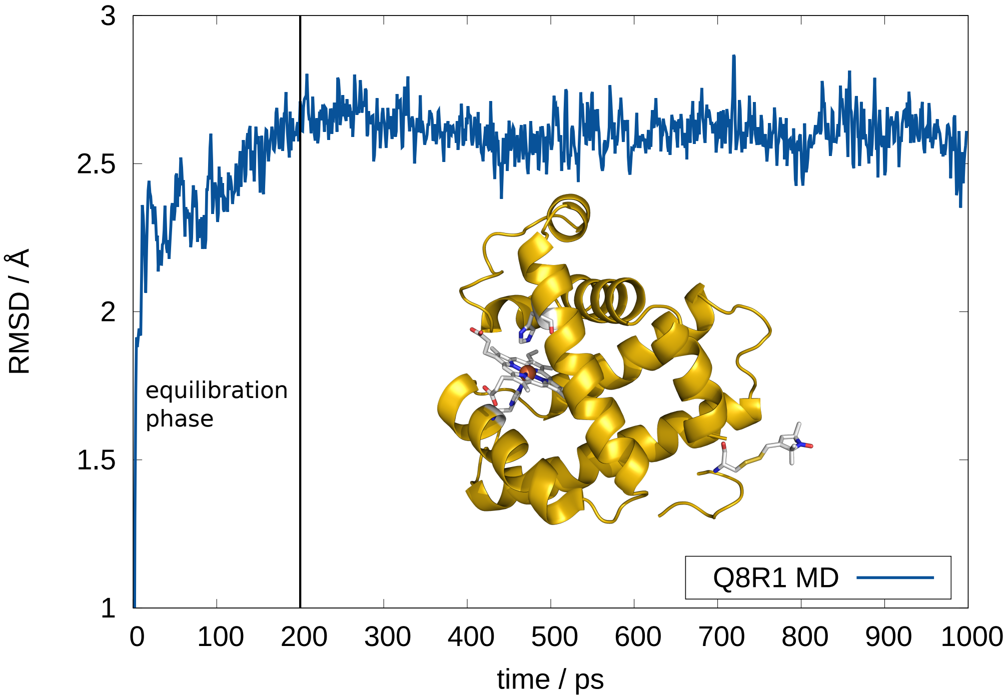

Figure S4. RMSD of Q8R1 as a function of time The first 200 ps are chosen as the equilibration phase and not considered for further structural evaluation.

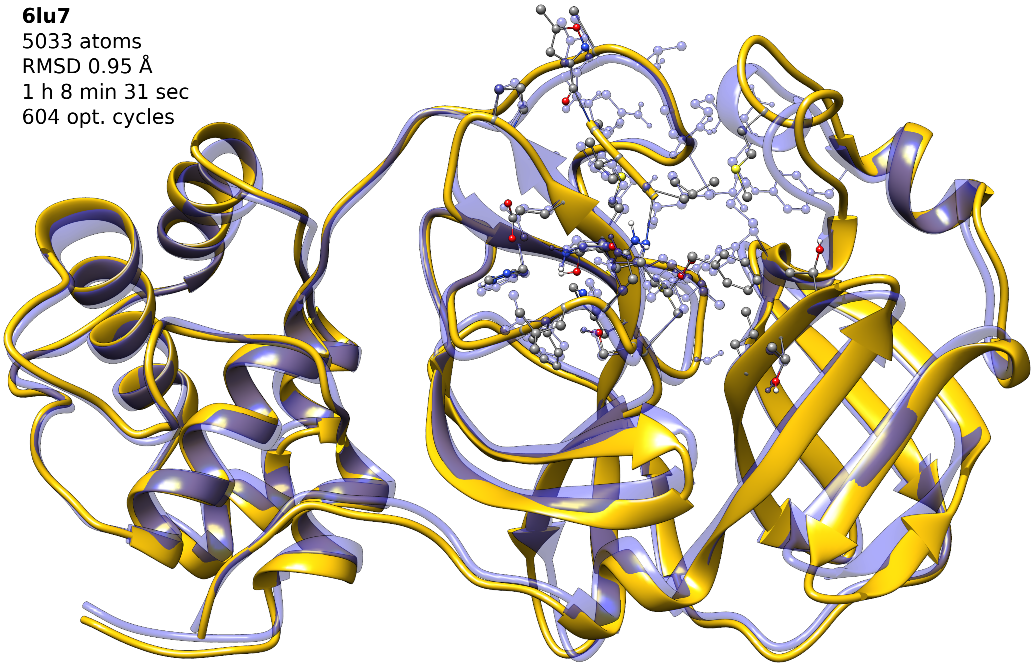

Figure S5. Geometry optimization of the COVID-19 main protease in complex with an inhibitor N3. Within 1 hour and 622 optimization cycles on four Intel© Xeon CPUs, a stationary point on the PES is found. The RMSD between the experimental and the GFN-FF structure is 0.95 Å. A GBSA(H_2O) solvation model was used. The structure is taken from Ref.[31]

Table S1: Organic protein benchmark set. Average deviations of four types of dihedral angles (in degree) for the test set of 70 protein structures as well as average C_α and heavy atom (ha) RMSD (in Å) in each case with respect to the crystal structure.

	angles / °				RMSD / Å	
	$\Delta\phi$	$\Delta\psi$	$\Delta\xi$	$\Delta\omega$	C_α	ha
UFF	24.2	22.3	11.7	23.2	1.82	2.22
GFN2-xTB	14.2	14.7	10.1	5.4	0.59	0.87
AMBER*	19.8	15.3	12.1	5.0	0.69	0.97
OPLS2005	12.4	12.3	10.7	4.6	0.58	0.84
GFN-FF	12.4	11.4	9.4	7.5	0.60	0.81

Table S2: TMG145 benchmark set. Statistical evaluation of the performance in terms of bond lengths and angles of the TMG145 benchmark set. Bond lengths in pm and angles in degree.

	bonds(M-A) / pm			angles(A-M-B) / °		
	MD	MAD	SD	MD	MAD	SD
GFN2-xTB	2.1	8.3	15.1	-0.50	3.9	6.6
UFF	2.6	14.6	21.1	-0.26	8.4	12.0
GFN-FF	-2.4	9.7	14.7	-0.03	5.7	8.7

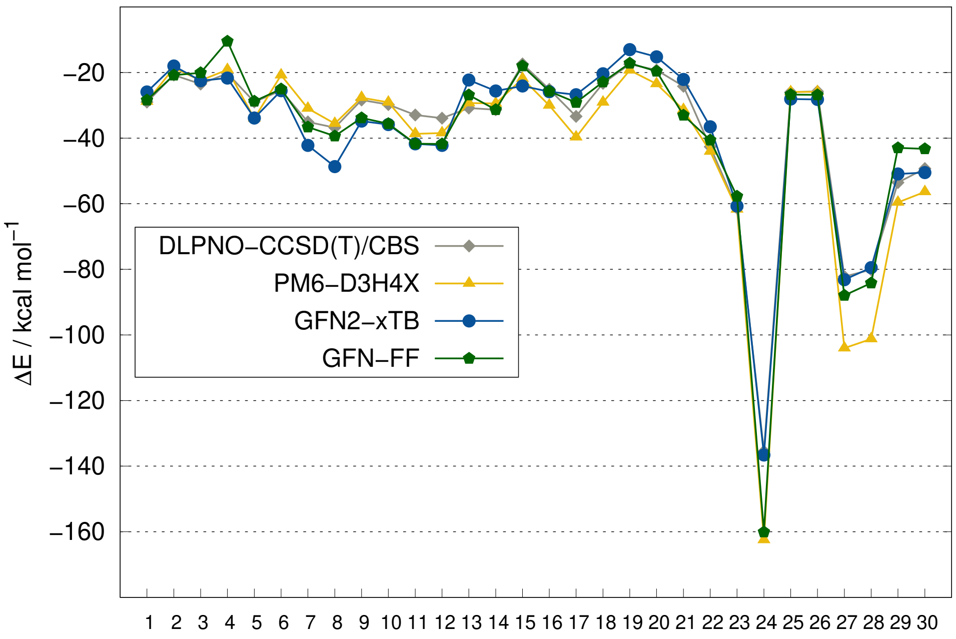

Figure S6. Performance on the S30L benchmark set. Association energies for all systems in the S30L set computed with GFN-FF, GFN2-xTB and PM6-D3H4X. The reference energies are obtained at the DLPNO-CCSD(T)/CBS level.

Table S3: Association energies of the S30L benchmark set. Values are computed with GFN-FF and semiempirical methods for 30 large noncovalent complexes containing only main group elements (S30L). The values are given in kcal mol^{-1}.

system	GFN-FF	GFN2-xTB	PM6-D3H4X	ref.
1	-28.41	-25.93	-28.83	-29.04
2	-20.81	-18.02	-18.63	-20.78
3	-20.07	-22.50	-22.41	-23.54
4	-10.47	-21.72	-19.18	-20.27
5	-28.78	-33.88	-33.96	-28.99
6	-25.05	-25.57	-20.83	-25.50
7	-36.64	-42.20	-30.95	-35.06
8	-39.38	-48.70	-35.57	-36.79
9	-33.84	-34.83	-27.66	-28.38
10	-35.60	-35.86	-29.12	-29.78
11	-41.72	-41.74	-38.69	-32.95
12	-41.78	-42.21	-38.45	-33.92
13	-26.86	-22.30	-29.32	-30.83
14	-31.36	-25.64	-29.45	-31.33
15	-18.01	-24.10	-21.86	-17.39
16	-25.72	-25.85	-30.03	-25.12
17	-29.12	-26.78	-39.73	-33.38
18	-22.88	-20.38	-29.11	-23.31
19	-17.16	-13.05	-19.32	-17.47
20	-19.64	-15.23	-23.48	-19.25
21	-33.02	-22.14	-31.35	-24.21
22	-40.62	-36.57	-44.06	-42.63
23	-57.76	-60.72	-61.72	-61.32
24 1	-160.1	-136.59	-162.49	-135.5
25	-26.75	-28.08	-25.95	-25.96
26	-26.82	-28.21	-25.89	-25.77
27	-87.95	-83.16	-104.03	-82.18
28	-84.20	-79.49	-101.22	-80.11
29	-42.98	-50.95	-59.62	-53.54
30	-43.28	-50.52	-56.39	-49.28

Table S4: Performance of different methods on the S30L benchmark. MAD of the association energies computed with GFN-FF and other QM methods for the S30L benchmark set. The values are given in kcal mol^{-1}.

method	S30L
GFN1-xTB	6.08
GFN2-xTB	4.05
GFN-FF	4.15
DFTB3-D3(BJ)	6.90
PM6-D3	6.78
PM6-D3H4X	5.15
HF-3c	5.20
B97-3c	3.20
PBEh-3c	2.60
B3LYP-D3	5.88
TPSS-D3	4.10
PBE-D3	3.10
SCAN-D4	2.00
PW6B95-D4	2.50

Table S5: MAD of the noncovalent interaction energies computed with GFN-FF and other semiempirical QM methods. Subsets taken from the GMTKN55 benchmark set. The values are given in kcal mol^{-1}.

benchmark	GFN0-xTB	GFN1-xTB	GFN2-xTB	PM7	GFN-FF
S22	1.630	1.330	0.758	0.768	0.89
S66	1.298	1.080	0.730	0.755	0.76
RG18	0.442	0.324	0.112	0.644	0.39
ADIM6	0.508	1.007	1.152	0.202	0.61
CARBHB12	2.316	0.670	1.084	1.883	1.81
HAL59	1.769	1.345	1.276	4.106	1.88
HEAVY28	0.908	0.658	0.608	2.948	0.77
PNICO23	2.000	2.331	1.104	5.673	1.98

Table S6: MAD of the conformational energies computed with GFN-FF and other semiempirical QM methods. Subsets taken from the GMTKN55 benchmark set. The values are given in kcal mol^{-1}.

benchmark	GFN0-xTB	GFN1-xTB	GFN2-xTB	PM6-D3H4X	GFN-FF
ACONF	0.80	0.66	0.19	0.46	0.13
Amino20x4	1.00	1.11	0.95	1.47	1.52
BUT14DIOL	0.81	0.95	1.25	1.49	1.12
ICONF	2.33	2.63	1.63	3.13	3.02
MCONF	1.66	1.44	1.72	1.42	0.48
PCONF21	1.79	2.17	1.76	2.58	1.60
SCONF	1.69	2.50	1.64	5.32	1.41
UPU23	1.34	1.24	2.90	2.38	2.95

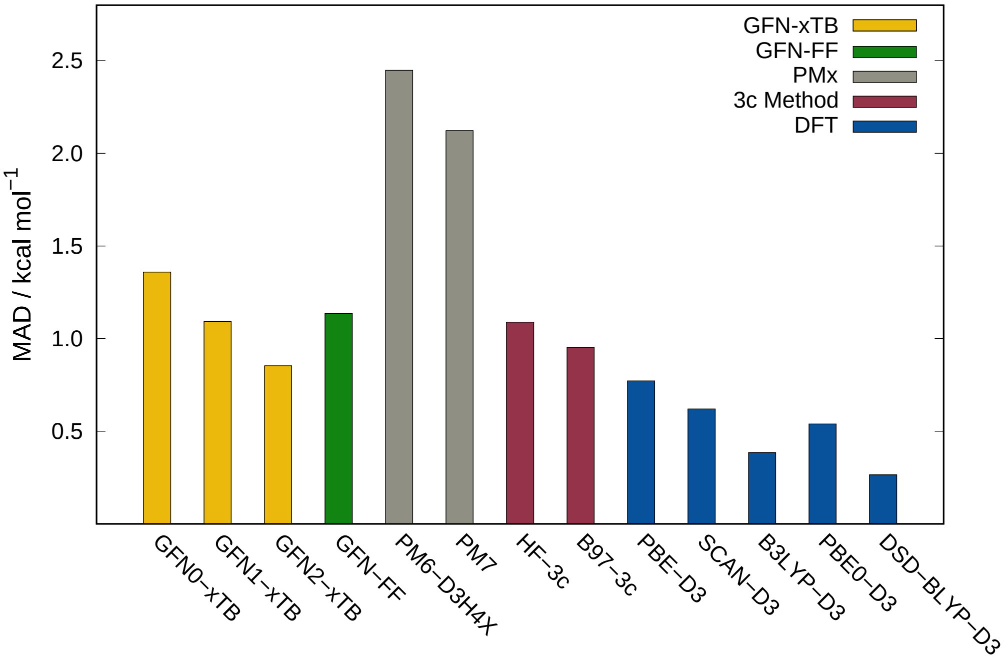

Figure S7. Noncovalent interaction energies from the GMTKN55. MAD of the noncovalent interaction energies computed with GFN-FF and other QM methods for subsets of the GMTKN55 benchmark set. The values are given in kcal mol^{-1}.

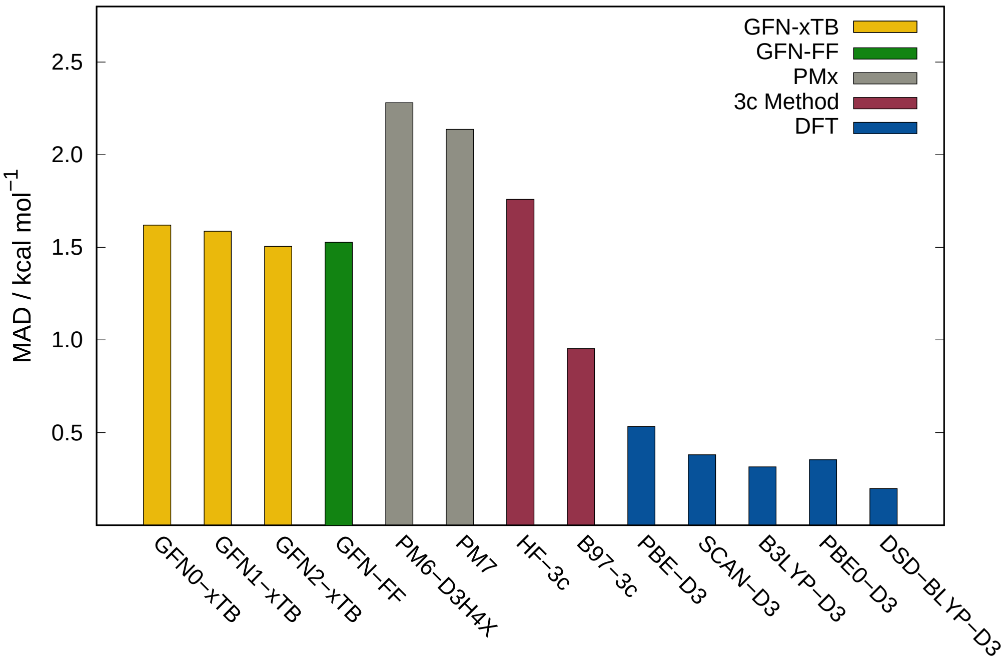

Figure S8. Conformational energies from the GMTKN55. Average MAD of the conformational energies computed with GFN-FF and other QM methods for subsets of the GMTKN55 benchmark set. The values are given in kcal mol^{-1}.

Table S7: Average MAD of the noncovalent and conformational energies for the GMTKN55 benchmark set. Values are computed with GFN-FF and other QM methods and are given in kcal mol^{-1}.

method	GMTKN55 noncovalent interactions	GMTKN55 conformational energies
GFN0-xTB	1.36	1.62
GFN1-xTB	1.09	1.59
GFN2-xTB	0.85	1.51
GFN-FF	1.13	1.53
PM6-D3H4X	2.45	2.28
PM7	2.12	2.14
HF-3c	1.09	1.76
PBE-D3	0.77	0.53
SCAN-D3	0.62	0.38
B97-3c	0.95	0.47
B3LYP-D3	0.39	0.32
PBE0-D3	0.54	0.35
DSD-BLYP-D3	0.27	0.20

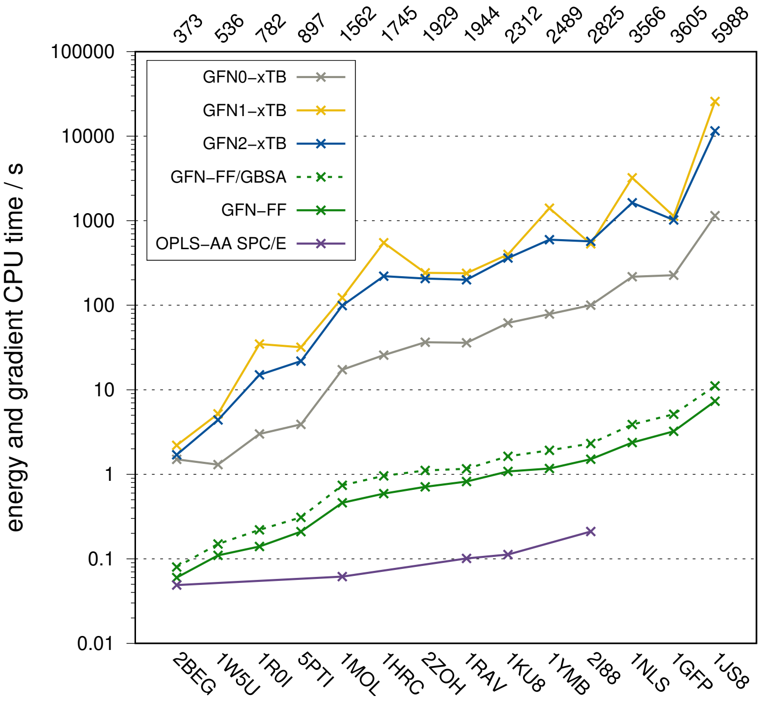

Figure S9. Timings for energy and gradient computation of different GFN methods. CPU times (given in seconds) for single point energy/gradient calculations of 14 proteins. GBSA(H_2O) had to be used for GFN1-xTB and GFN2-xTB in order to achieve SCC convergence. This is not necessary for GFN0-xTB and GFN-FF. Thus, for GFN-FF the timings including the GBSA model are shown separately. Additionally the timings for OPLS-AA SPC/E with counter ions under periodic boundary conditions are shown for those proteins, where parameters are available. The PDB identifiers are given on the bottom x-axis, the corresponding number of atoms is given on top. Computations were performed on a single core of a quad-core desktop machine with 4.20 GHz Intel© i7-7700K CPUs.

Section S9. The description of water

The performance of GFN-FF for small to medium sized clusters is shown in Figure S10. In part A, the dissociation curve of the water dimer is shown and compared to accurate CCSD(T)/CBS reference values.[32] Energies are shown relative to the equilibrium structure. GFN-FF shows the energetic minimum at the same inter-monomer distance as the reference, indicating the correct length of the hydrogen bond. For the water dimer the interaction energy is semi-quantitatively correct but slightly overestimated at larger distances with GFN-FF. In part B dissociation energies of small to medium sized neutral water cluster are shown, taken from the WATER27 benchmark set.[33] The larger clusters are slightly underbound

by GFN-FF but the error is in all cases <10% of D_e which is difficult to achieve even with sophisticated DFT methods. The GFN2-xTB method shown for comparison performs exceptionally well for this set. The errors of the UFF competitor method (not shown) exceed hundreds of kcal/mol for this benchmark set.

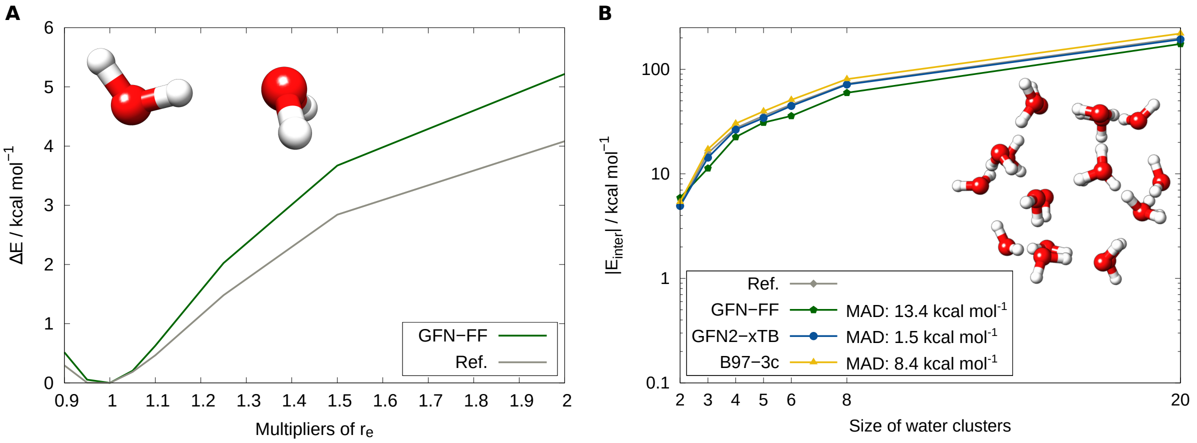

Figure S10. Performance of GFN-FF for the description of water clusters. A: Dissociation curve of the water dimer at different inter-monomer separations, where the equilibrium distance r_e is multiplied by the scaling factor shown (taken from revised S66x8 set, see Ref. 32). **B**: Performance of different methods on the neutral complexes of the WATER27 benchmark set. Given are the cluster binding energies on a logarithmic scale and the corresponding MAD values compared to the CCSD(T)/CBS reference data shown as solid grey line.

For a water cluster consisting of 1451 molecules a MD simulation at 298 K was performed for 5 ps with an equilibration time of the same length. An average a density of 1.13 $\pm$ 0.003 g cm^{-1} is obtained. The deviation of 0.13 g cm^{-1} from the well known experimental value corresponds to an average underestimation of the intermolecular distances by about 4 %. However, this is not seen for the water dimer as depicted in Figure S10, where the inter-monomer distance corresponds well with the reference.

Section S10. Thermostatistical corrections

GFN-FF computed zero-point vibrational energies (ZPVE) and total molecular free energies at 298 K (G_{298}) are compared to corresponding values at the low-cost B97-3c DFT theoretical level. Figure S11 shows a comparison of ZPVE values at B97-3c and GFN2-xTB values for

a set of 39 medium sized organic molecules taken from a benchmark study of Li *et al.*[34]

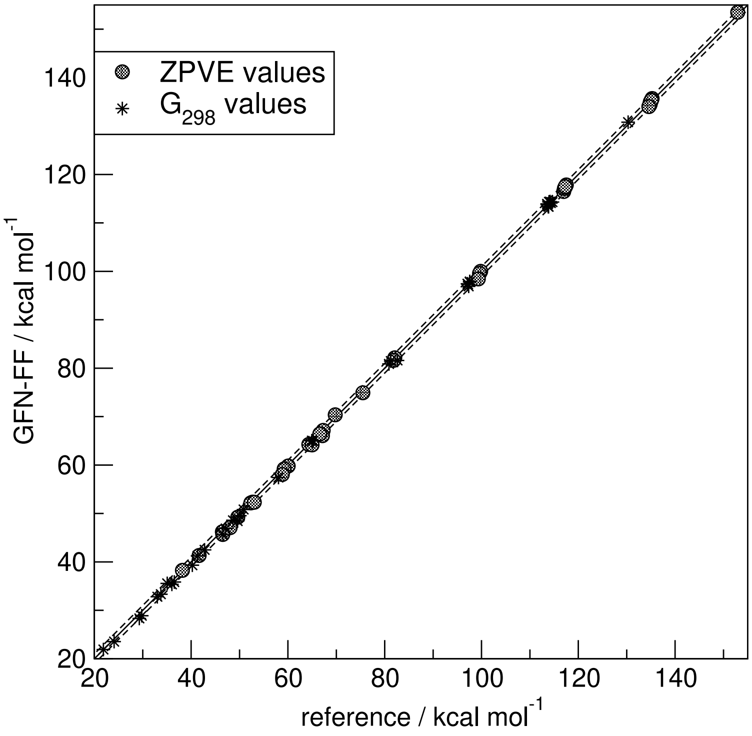

Figure S11. Comparison of GFN-FF thermostatistical data with corresponding B97-3c DFT reference values. The set consists of 39 organic molecules ranging from ethane (smallest) to n-octane (largest). The solid line shows the one-to-one correspondence and the dashed ones indicate a common error range for chemical accuracy, i.e., ±1 kcal mol^{-1}.

The data have been used to determine as usual a frequency scale factor for GFN-FF. An optimum value of 1.03 was found and should be used as default in corresponding vibrational or thermostatistical applications. The scaled frequencies are listed in the output of the `xtb` program. As can be clearly seen from the graph, there is a very good reproduction of the DFT reference thermostatistical properties by GFN-FF. The MAD for the ZPVE data is only 0.37 kcal mol^{-1} (MD=0.20 kcal mol^{-1}). In actual applications where normally differences of the values for reactants and products are taken, the effective error is may be even smaller because of cancellation. The performance for the free energies is similar with an MAD of only 0.42 kcal mol^{-1} (MD=0.22 kcal mol^{-1}).

References

[1] "Semiempirical Extended Tight-Binding Program Package `xtb`", https://github.com/grimme-lab/xtb. Accessed: 2020-04-20.

[2] S. Grimme, A. Hansen, *Angew. Chem. Int. Ed.* **2015**, *54*(42), 12308–12313.

[3] T. A. Halgren, *J. Comput. Chem.* **1996**, *17*(5-6), 490–519.

[4] S. Grimme, *J. Chem. Theory Comput.* **2014**, *10*(10), 4497–4514.

[5] P. Pyykkö, M. Atsumi, *Chem. Eur. J.*, *15*, 186–197, DOI 10.1002/chem.200800987.

[6] S. Grimme, C. Bannwarth, P. Shushkov, *J. Chem. Theory Comput.* **2017**, *13*(5), 1989–2009.

[7] B. Axilrod, E. Teller, *J. Chem. Phys.* **1943**, *11*(6), 299–300.

[8] Y. Muto, *J. Phys. Math. Soc. Jpn.* **1943**, *17*, 629–631.

[9] E. Caldeweyher, S. Ehlert, A. Hansen, H. Neugebauer, S. Spicher, C. Bannwarth, S. Grimme, *J. Chem. Phys.* **2019**, *150*, 154122.

[10] R. W. Floyd, *Commun. ACM* **1962**, *5*(6), 345.

[11] M. Mantina, R. Valero, C. J. Cramer, D. G. Truhlar, *CRC Handbook of Chemistry and Physics* **2013**, *94*.

[12] S. Grimme, J. Antony, S. Ehrlich, H. Krieg, *J. Chem. Phys.* **2010**, *132*, 154104.

[13] S. Grimme, S. Ehrlich, L. Goerigk, *J. Comput. Chem.* **2011**, *32*, 1456–1465.

[14] D. C. Ghosh, N. Islam, *Int. J. Quant. Chem.* **2010**, *110*(6), 1206–1213.

[15] E. R. Johnson, A. D. Becke, *J. Chem. Phys.* **2005**, *123*, 024101.

[16] C. Bleiholder, D. B. Werz, H. Köppel, R. Gleiter, *J. Am. Chem. Soc.* **2006**, *128*(8), 2666–2674.

[17] C. Bleiholder, R. Gleiter, D. B. Werz, H. Köppel, *Inorg. Chem.* **2007**, *46*(6), 2249–2260.

[18] L. A. Curtiss, K. Raghavachari, P. C. Redfern, J. A. Pople, *J. Chem. Phys.* **1997**, *106*(3), 1063–1079.

[19] K. Levenberg, *Q. Appl. Math.* **1944**, *2*, 164–168.

[20] D. Marquardt, *J. Soc. Ind. Appl. Math.* **1963**, *11*, 431–441.

[21] S. Grimme, J. G. Brandenburg, C. Bannwarth, A. Hansen, *J. Chem. Phys.* **2015**, *143*(5), 054107.

[22] J. G. Brandenburg, C. Bannwarth, A. Hansen, S. Grimme, *J. Chem. Phys.* **2018**, *148*(6), 064104.

[23] C. Bannwarth, S. Ehlert, S. Grimme, *J. Chem. Theory Comput.* **2019**, *15*(3), 1652–1671.

[24] J. G. Brandenburg, S. Grimme, *Top. Curr. Chem.* **2014**, *345*, 1–23.

[25] P. S. Brahmkshatriya, P. Dobeš, J. Fanfrlík, J. Řezáč, K. Paruch, A. Bronowska, M. Lepšík, P. Hobza, *Curr. Comput.-Aid. Drug.* **2013**, *9*, 118–129.

[26] J. J. P. Stewart, *J. Mol. Model.* **2013**, *19*, 1–32.

[27] J. J. P. Stewart, *MOPAC2016*, Stewart Computational Chemistry, Colorado Springs, CO, USA, **2016**, http://OpenMOPAC.net (August 16, 2016).

[28] F. Furche, R. Ahlrichs, C. Httig, W. Klopper, M. Sierka, F. Weigend, *WIREs Comput. Mol. Sci.* **2014**, *4*, 91–100.

[29] R. Ahlrichs, M. Bär, M. Häser, H. Horn, C. Kölmel, *Chem. Phys. Lett.* **1989**, *162*, 165–169.

[30] F. Neese, *WIREs Comput. Mol. Sci.* **2012**, *2*, 73–78.

[31] X. Liu, B. Zhang, Z. Jin, H. Yang, Z. Rao, **2020**, 10.2210/pdbluU7/pdb.

[32] B. Brauer, M. K. Kesharwani, S. Kozuch, J. M. Martin, *Phys. Chem. Chem. Phys.* **2016**, *18*(31), 20905–20925.

[33] V. S. Bryantsev, M. S. Diallo, A. C. van Duin, W. A. Goddard III, *J. Chem. Theory Comput.* **2009**, *5*(4), 1016–1026.

[34] Y.-P. Li, A. T. Bell, M. Head-Gordon, *J. Chem. Theory Comput* **2016**, *12*(6), 2861–2870.
